# Using Mendelian Randomisation methods to understand whether diurnal preference is causally related to mental health

**DOI:** 10.1038/s41380-021-01157-3

**Published:** 2021-06-08

**Authors:** Jessica O’Loughlin, Francesco Casanova, Samuel E. Jones, Saskia P. Hagenaars, Robin N. Beaumont, Rachel M. Freathy, Edward R. Watkins, Céline Vetter, Martin K. Rutter, Sean W. Cain, Andrew J. K. Phillips, Daniel P. Windred, Andrew R. Wood, Michael N. Weedon, Jessica Tyrrell

**Affiliations:** 1 Genetics of Complex Traits, The College of Medicine and Health, University of Exeter, The RILD Building, RD&E Hospital, Exeter, UK; 2 Social, Genetic and Developmental Psychiatry Centre, Institute of Psychiatry, Psychology and Neuroscience, King’s College London, London, UK; 3 Psychology, Mood Disorders Centre, College of Life and Environmental Sciences, University of Exeter, Exeter, UK; 4 Circadian and Sleep Epidemiology Laboratory, Department of Integrative Physiology, University of Colorado Boulder, Boulder, CO USA; 5 Faculty of Biology, Medicine and Health, The University of Manchester, Manchester, UK; 6 Manchester Diabetes Centre, Manchester University NHS Foundation Trust, Manchester Academic Health Sciences Centre, Manchester, UK; 7 Turner Institute for Brain and Mental Health, School of Psychological Sciences, Monash University, Clayton, VIC Australia

**Keywords:** Genetics, Depression

## Abstract

Late diurnal preference has been linked to poorer mental health outcomes, but the understanding of the causal role of diurnal preference on mental health and wellbeing is currently limited. Late diurnal preference is often associated with circadian misalignment (a mismatch between the timing of the endogenous circadian system and behavioural rhythms), so that evening people live more frequently against their internal clock. This study aims to quantify the causal contribution of diurnal preference on mental health outcomes, including anxiety, depression and general wellbeing and test the hypothesis that more misaligned individuals have poorer mental health and wellbeing using an actigraphy-based measure of circadian misalignment. Multiple Mendelian Randomisation (MR) approaches were used to test causal pathways between diurnal preference and seven well-validated mental health and wellbeing outcomes in up to 451,025 individuals. In addition, observational analyses tested the association between a novel, objective measure of behavioural misalignment (Composite Phase Deviation, CPD) and seven mental health and wellbeing outcomes. Using genetic instruments identified in the largest GWAS for diurnal preference, we provide robust evidence that early diurnal preference is protective for depression and improves wellbeing. For example, using one-sample MR, a twofold higher genetic liability of morningness was associated with lower odds of depressive symptoms (OR: 0.92, 95% CI: 0.88, 0.97). It is possible that behavioural factors including circadian misalignment may contribute in the chronotype depression relationship, but further work is needed to confirm these findings.

## Introduction

Circadian rhythms are approximately 24-h cyclical physiological processes found in most living organisms, including humans [1]. Individuals are often classified by their diurnal preference as morning people (“larks”), who prefer going to bed earlier and waking earlier, or evening people (“owls”) who prefer a later bedtime and waking later [2]. Diurnal preference (morning or evening preference) can be considered a behavioural manifestation of the circadian system, sometimes referred to as chronotype.

There is evidence that an individual’s diurnal preference is linked to disease development, including psychiatric disorders, with numerous cross-sectional studies reporting that early-type individuals have a lower risk of depressive symptoms, diagnosed depression and antidepressant use [3–6]. A prospective study of 32,000 middle-aged women demonstrated early types had a lower risk of developing depression when compared to intermediate types, even after extensive confounder adjustment and exclusion of subgroups known to experience higher levels of circadian misalignment (e.g., shift workers or short sleepers) [7]. Additional studies have shown eveningness to associate with increased anxiety with some evidence that the relationship is present in women only [8, 9]. However, these results have the potential to be confounded and may be influenced by reverse causality.

**Fig. 1:**
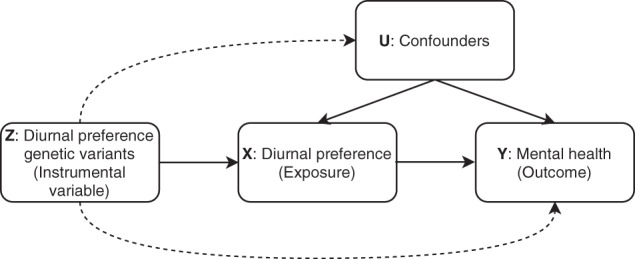
The principles of Mendelian Randomisation. The key assumptions are (1) *Z*, the instrumental variable, should be robustly associated with the exposure *X*; (2) *Z* should not be associated with *U* (confounders) of the *X*-*Y* association; (3) the only path from *Z* to the outcome (*Y*) is through *X*. The dotted lines represent violations of the assumptions (2) and (3).

Genetic techniques can help us to test whether causal relationships exist between diurnal preference and mental health outcomes. A recent genome-wide association study (GWAS) identified 351 genetic variants associated with diurnal preference with an estimated heritability of 13.7% [10]. That study utilised a technique known as Mendelian Randomisation (MR; Fig. 1) to provide evidence that morningness was causally associated with higher subjective wellbeing and lower odds of schizophrenia and current depressive symptoms (a relatively simple measure defined using the responses to two self-report questions available in 105,739 UK Biobank participants) [11]. However, this study did not explore more detailed measures of mental health, nor test whether the results were sex-specific, as suggested by the observational literature.

In addition to associations between diurnal preference and mental health outcomes, there is a growing body of evidence suggesting that the misalignment between an individual’s sleep-wake cycle and their endogenous circadian rhythms might contribute to adverse mental and physical health [12]. Societal determination of work time and free time can interfere with an individual’s diurnal preference [13–15]. For example, evening people (late preference) experience this mismatch when they are forced to wake early for work, while early types might be forced to stay up longer on weekends to adhere with social norms [16]. This phenomenon has been coined “social jetlag”, and can be quantified by calculating the shift in sleep patterns (in hours) between work and free days [17]. This misalignment measure can be derived from questionnaire data, and has been associated with seasonal depression and depressive symptoms [18–20]. However, these studies lack well-defined mental health outcomes and quantitative measures of behavioural misalignment.

**Fig. 2:**
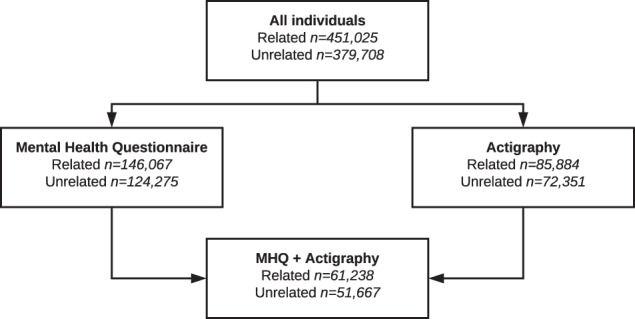
Flowchart illustrating the number of individuals used in these analyses. All numbers represent UK Biobank participants of European ancestry.

Here, we use genetic techniques to test the role of diurnal preference on mental health outcomes in up to 451,025 individuals of European ancestry in the UK Biobank study. We utilise the data from the mental health questionnaire (MHQ) (available in 146,067; Fig. 2), to derive *clinically relevant* depression and anxiety measures, as well as measures of general wellbeing. Second, we utilise data from a subset of individuals in the UK Biobank with actigraphy monitoring (available in 85,884; Fig. 2) to derive an objective actigraphy-based measure of behavioural misalignment (i.e., Composite Phase Deviation; CPD), enabling us to test whether an individual’s diurnal preference is associated with misalignment [12]. Circadian misalignment as defined and quantified by CPD represents a gene-by-environment (G × E) interaction (with G being captured by diurnal preference). A genetic instrument for CPD would be equivalent to the diurnal preference instrument (Supplementary Fig. 1). Here we use CPD to investigate the behavioural element of variable sleep timing on mental health and wellbeing using observational models to test the hypothesis that more misaligned individuals have poorer mental health and wellbeing.

## Methods

### UK Biobank

The UK Biobank is a health resource with extensive phenotypic and genetic data available for over 500,000 participants, who were aged between 40 and 70 at recruitment (from 2006 to 2010). Participants were recruited from across the UK and attended one of 22 centres in England, Scotland and Wales, to provide detailed sociodemographic, health and anthropometric data as well as providing blood and urine samples for subsequent analyses. Participants consented to having their health followed and many have subsequently participated in further monitoring or completed additional questionnaires. The cohort is described in detail elsewhere [21].

Genetic data were available for all individuals and this data underwent extensive central quality control [22]. We used data on up to 451,025 European individuals from the full UK Biobank data release that had genetic data available. Europeans were defined by performing principal component analysis in the 1000 Genomes (1KG) reference panel using a subset of variants that were of high quality in the UK Biobank. We then used these loadings to project all the UK Biobank samples into the same principal component space and used a k-means clustering approach to define a European cluster using principal components 1–4. Of all included individuals, 146,067 had completed the MHQ, 85,884 had completed the actigraphy monitoring and 61,238 had both available (Fig. 2) [23, 24].

We also defined a subset of unrelated individuals, using the King Kinship matrix to exclude individuals related up to third degree. Ancestral principal components were then generated within these individuals for subsequent analyses. Within our unrelated subset, we had data on up to 379,708 individuals (124,275 with the MHQ, 72,351 with actigraphy monitoring and 51,667 with both) (Fig. 2).

### Exposure and outcome measures

Detailed information on the exposure and outcome measures used in this study are available in the online supplement. They are described briefly below.

### Exposures

#### Diurnal preference

Diurnal preference was self-reported in UK Biobank (data field 1180). Participants were asked “Do you consider yourself to be?” with one of six possible answers: “Definitely a ‘morning’ person”, “More a ‘morning’ than ‘evening’ person”, “More an ‘evening’ than a ‘morning’ person”, “Definitely an ‘evening’ person”, “Do not know” or “Prefer not answer”, this was then coded as 2, 1, −1, −2, 0 and missing, respectively. We then defined a binary morning person phenotype, where we coded participants reporting to be “More an ‘evening’ than a ‘morning’ person” or “Definitely an ‘evening’ person” as 0 (controls) and those answering “Definitely a ‘morning’ person” or “More a ‘morning’ than ‘evening’ person” as 1 (cases). All other responses were coded to missing. This single-item measure of diurnal preference correlates well with the overall score of the Morningness–Eveningness Questionnaire (*r* = 0.72) [25, 26], sleep timing [27] and dim light melatonin onset [28].

#### Behavioural circadian misalignment

**Fig. 3:**
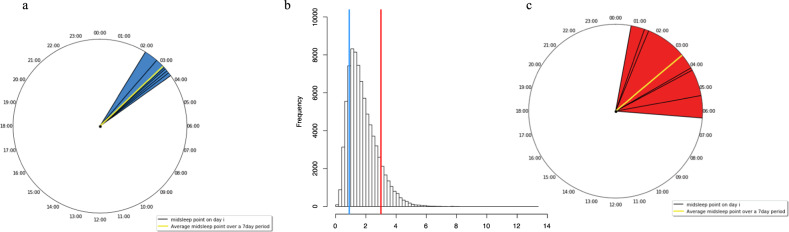
A diagram to illustrate Composite Phase Deviation (CPD). **b** The frequency distribution of CPD in 76,334 UK Biobank participants. The vertical blue line represents an individual with lower CPD (0.90) compared to the red line which represents higher CPD (3.0). The midsleep points over a 7-day period (i_1-7_) of these individuals are shown. **a** The individual with low CPD i.e. low circadian misalignment. The yellow line represents the average midsleep point over the 7 days and the blue shaded area shows the deviation of midsleep around the average. **c** The individual with high CPD i.e. greater circadian misalignment. The yellow line represents the average midsleep point over the 7 days and the red shaded area shows the deviation of midsleep around the average.

Circadian misalignment has been derived using actigraphy data in the UK Biobank by computing the CPD metric (online supplement) [12]. Briefly, CPD combines the deviation of each night’s sleep midpoint from both the individual’s average sleep midpoint and the previous night’s sleep midpoint. CPD captures both the overall variability in sleep timing and changes in sleep timing between consecutive nights. A higher CPD value has been proposed to capture greater misalignment (Fig. 3).

### Outcomes

A range of mental health and wellbeing measures were derived in the UK Biobank. The derivation of these measures is explained briefly below, with detailed information provided in the online supplement.

### Definitions of depression

#### Depressive symptoms

The ‘depressive symptoms’ measure has been defined in the whole UK Biobank as previously described [29]. Briefly, indivduals were considered a case if they met one or more of the following:

- self-reported seeing a GP for nerves/anxiety or depression AND reported at least a 2-week duration of depression or unenthusiasm;
- self-reported seeing a psychiatrist for nerves/anxiety or depression AND reported at least a 2-week duration of depression or unenthusiasm;
- had the following ICD-10 codes in the Hospital Episode Statistics: F33 representing recurrent major depressive disorder (MDD) or F32 representing single-episode MDD.

From this, we defined a binary lifetime depressive symptoms variable including 49,076 cases and 292,984 controls.

#### Major depression

We defined ‘major depression’ in a subset of individuals in the UK Biobank with MHQ data available, which utilises the well-established and validated Composite International Diagnostic Interview (CIDI) [30]. We excluded any depression-related phenotypes including self-report psychosis and mania. Using this definition, proposed by Davis et al., we defined a binary variable in 41,691 cases and 104,346 controls and a continuous CIDI severity score ‘CIDI severity’ (online supplement).

#### Current severity of depression

Current severity of depression was defined using the Patient Health Questionnaire (PHQ)9, a well-validated measure of current depression [31]. Respondents were asked how much each item (symptom) had bothered them over the past 2 weeks with the options to respond: “not at all”, “several days”, “more than half the days”, and “nearly every day”, scored as 0, 1, 2, and 3, respectively. We created a continuous current depression severity measure from the PHQ9 from 0 to 27 with higher scores representing more severe depression (‘PHQ9 severity’) (online supplement).

### Wellbeing

#### Wellbeing score

A wellbeing score was derived from three variables that made up part of the mental health questionnaire. All participants completing the MHQ were asked: “In general how happy are you?” (data field 20458) and “In general how happy are you with your health?” (data field 20459), with the options to respond “Extremely unhappy”, “Very unhappy”, “Moderately unhappy”, “Moderately happy”, “Very happy”, “Extremely happy”, “Do not know” and “Prefer not to answer”. We recoded these variables to a scale from 1–6 with 1 representing extremely unhappy and 6 representing extremely happy. Participants who preferred not to answer or did not know were set to missing. Participants were also asked: “To what extent do you feel your life to be meaningful?” with the options “Not at all”, “A little”, “A moderate amount”, “Very much”, “An extreme amount”, “Do not know” and “Prefer not to answer”. This variable was scaled from 1 to 5 where 1 represented not at all and 5 an extreme amount. These three variables were summed to provide the overall ‘wellbeing score’. This was available for 141,829 participants with valid genetic data.

### Anxiety

#### General anxiety disorder

A binary variable representing general anxiety disorder was derived from the Generalised Anxiety Disorder Composite International Diagnostic Interview GAD-CIDI (online supplement). We defined 7244 GAD cases with 89,665 controls (GAD). Further, we defined a continuous current GAD severity score using the GAD7 questionnaire (GAD severity) (online supplement).

### Genetic variants for diurnal preference

We selected 351 variants, identified in the most recent GWAS of diurnal preference, from UK Biobank’s imputation dataset. Variants were excluded if the genotype probability was <0.9. The variants were weighted by the effect on diurnal preference excluding UK Biobank (Supplementary Table 1). A smaller subset of 108 variants was also selected that were associated with diurnal preference at genome-wide significance in the 23andMe only data for subsequent 2-sample MR analyses, to reduce the impact of Winner’s curse (Supplementary Table 2) [32].

These variants were utilised to create a weighted genetic risk score (GRS; Eq. 1). First, variants were recoded to represent the number of morningness alleles. Each variant was then weighted by its effect size. The GRSs for diurnal preference were robustly associated with self-reported diurnal preference in the UK Biobank, explaining 5.4% of the variance (and 1.8% for the GRS with the smaller subset of 108 SNPs that were identified using 23&Me Only).

1 $$\begin{array}{l}{\rm{GRS}}_{\rm{w}} = \beta _1d_1 + \beta _2d_2 + \ldots + \beta _nd_n = \mathop {\sum }\limits_{i = 1}^n \beta _id_i\\ {\rm{GRS}}_{\rm{s}} = \frac{{n \times {\rm{GRS}}_{\rm{w}}}}{{\mathop {\sum }\nolimits_{i = 1}^n \beta _i}}\end{array}$$

where *β*_*i*_ represents the effect size and *d*_*i*_ represents the effect allele dosages for variant *i* of *n*, and *GRS*_w_ and *GRS*_s_ represent the weighted and standardised GRSs, respectively.

### Data analysis

#### Observational associations between diurnal preference and mental health measures

The mental health measures were regressed against the binary diurnal preference variable using logistic or ordinal models depending on the outcome (logistic: depressive symptoms, major depression and generalised anxiety, ordinal: CIDI severity, PHQ9 severity GAD severity and wellbeing score). We adjusted these for age, sex, and assessment centre and then further adjusted for the socioeconomic position (using the Townsend deprivation index, (TDI; variable 189 in UK Biobank)), smoking status (coded as never, former and current; variable 20116 in UK Biobank) and BMI (as calculated from weight/height^2^; variable 21001 in UK Biobank). These models were run in all individuals and in males and females separately.

#### Mendelian randomisation to test causal relationships between diurnal preference and mental health

Several MR approaches were employed, first, the standard one-sample instrumental variable analyses using the GRS_s_ was performed in the unrelated subset [33]. One-sample MR uses one dataset in the instrumental variable analysis to yield the causal estimate of the risk factor (here diurnal preference) on the outcome (here depression). This method enables sensitivity analyses to be easily performed but requires the unrelated sample as it cannot account for relatedness in the model. One-sample MR uses the two-stage least-squares regression estimator to predict the levels of morningness per genotype and then regress the mental health outcome against the predicted value. First, an unconfounded estimate of diurnal preference variation was estimated by taking the association between being a morning person and the diurnal preference GRS. The mental health outcome was then used as the dependent variable in a logistic regression (binary outcome) or ordered logistic (ordinal outcome) model.

Second, we investigated the causal relationship using two-sample MR (this uses two different study samples to estimate the instrument-risk factor and instrument-outcome associations) in the larger group of related individuals. The variants were extracted from our UK Biobank BOLT-LMM [34] GWAS summary data for the mental health outcomes. The variant-chronotype associations were taken from the primary GWAS of diurnal preference with the betas for both the 339 and 108 coming from 23&Me. Four different two-sample MR methods were used that follow different assumptions. Inverse variance weighted (IVW) MR [35] is a weighted regression of the chronotype variant-chronotype association against the chronotype variant-mental health/wellbeing association, with the intercept constrained to zero. Using the multiplicative random-effects IVW model accounts for balanced horizontal pleiotropy. However, further methods were performed to help account for horizontal pleiotropy. These included the MR-Egger analyses [36], which essentially performs the weighted regression without a constrained intercept, therefore allowing for unbalanced horizontal pleiotropy. MR-Egger assumes that the pleiotropic effects are independent of the variant-exposure effects (InSIDE assumption) and therefore weighted median MR which is robust to horizontal pleiotropy and does not rely on the InSIDE assumption was also used. This approach bases the overall estimate on the weighted median variant estimate [37], but does require that 50% or more of the instruments are valid. Finally, a penalised weighted median was calculated where outlying variants are penalised.

The results from MR analyses may represent a valid causal effect estimate under the condition of three core assumptions:

1. The genetic instrument needs to robustly associate with the exposure (‘relevance’);
2. There should be no joint causal influence affecting the exposure instrument and the outcome (‘independence’);
3. The instrument must not affect the outcome through any mechanism other than through the exposure (‘exclusion restriction’).

Using the MR power calculator (https://shiny.cnsgenomics.com/mRnd/) we have demonstrated at *p* = 0.05 with our sample size (for depressive symptoms) we have >99% power [38].

#### Mendelian randomisation to test the causal relationship between diurnal preference and circadian misalignment

The same MR methods were then utilised to test the causal relationship between diurnal preference and the downstream behavioural misalignment measure, CPD. A smaller subset of SNPs were used (*N* = 268) for the two-sample MR using the variants and effect sizes identified in the UKB/23andMe meta-analysis (that excluded actigraphy samples) as the exposure and CPD in UKB actigraphy as the outcome. As we are using UKB as one of our GWAS discovery samples, we exclude the actigraphy samples to avoid Winner’s curse. Including these samples would exaggerate the instrument-risk factor effect and potentially underestimate the instrument-outcome effect [39].

#### Observational associations between CPD and mental health measures

Logistic or ordinal logistic regression models were used to test the observational associations between CPD and the mental health/wellbeing outcomes. Models were adjusted for age at actigraphy, sex and season of actigraphy wear.

#### Doubling of the genetic risk

The one-sample and two-sample MR genetic associations with the exposure have been estimated using logistic regression and therefore yield a log odds ratio (ln(OR)) representing a change in the mental health outcome per change in diurnal preference on a log odds scale [40]. A unit increase on the log odds scale represents a LN [2] multiplicative increase in the odds of the outcome variable. Therefore, for interpretation, the average change in the mental health outcome per doubling in the genetic risk for morningness has been calculated.

### Sex-specific differences

All analyses were performed in all individuals and in males and females separately. To test the hypothesis that effects of diurnal preference and circadian misalignment on mental health differ in males and females, sex-specific effects were explored using the Fisher’s *z*-score method (Eq. 2).

2 $$z = \frac{{\beta _{\rm{m}} - \beta _{\rm{f}}}}{{\sqrt {SE_{\rm{m}}^2 + SE_{\rm{f}}^2} }}$$

### Sensitivity analyses

Several sensitivity analyses were performed. First, we stratified our analyses by shift worker status to test (a) whether diurnal preference demonstrated different effects on mental health and wellbeing in shift workers and (b) if shift workers were more misaligned. Second, the questionnaires used to derive the depression and anxiety measures, included items on sleep disturbances, which may cause overlap between the predictor and outcome and cause inflation of results. To test this we removed any individuals reporting that their sleep had changed (CIDI) or that they had trouble falling/ staying asleep (PHQ9 and GAD7). Third, the CPD analyses were stratified by (a) sex as women are more likely to report mental health problems [29] and (b) age (above and below 65: the presumed retirement age, based on the assumption that retired individuals should be more aligned). In addition, we adjusted the analyses for diurnal preference and the 351-variant diurnal preference GRS, to test the hypothesis that increased misalignment is associated with poorer mental health and wellbeing independent of chronotype. Furthermore, the primary CPD measure used the reference sleep midpoint as the mean sleep midpoint across all nights as the reference point (in place of ‘MSFsc’). We also used a secondary CPD measure, which utilises the mean sleep midpoint on “free” (Friday and Saturday) nights (MSFsc), with a correction applied for oversleeping on those nights [28, 41, 42] (online supplement). The original definition of CPD used sleep-duration-corrected free-night mean sleep midpoint (MSFsc) as this was more representative of unrestricted sleep timing [12]. However, only half of the UK Biobank participants with actigraphy data available also had data available in the employment history online follow-up. Hence, given the inability to differentiate between “restricted” and “unrestricted” nights of sleep, the assumption was made that “free nights” were Friday and Saturday night for all individuals. With the “free night” assumption in mind, it was decided that the primary CPD measure would use an all-night mean sleep midpoint and the secondary sensitivity measure would use MSFsc. The intention of using CPD with MSFsc as a secondary measure was to demonstrate that calculating CPD using the all-day average does not invalidate our findings by using a less ideal estimate of “natural” sleep timing, but one with much more data and thus less error. Finally, to assess whether the effect of misalignment on mental health and wellbeing were different in individuals taking sleep medications, antidepressants, antipsychotics or anxiolytics we stratified by medication use. ‘Not taking medication’ excluded any individuals reporting one or more of the medications (field 20003) at baseline.

## Results

There were 449,660 UK Biobank participants that had information on diurnal preference, of which 252,240 (62.6%) reported to be morning people. The demographics and mental health outcomes of morning and evening people are reported in Table 1. Generally, morning people were older, more likely to be female, had a lower BMI, were of higher SES (indicated by low TDI) and were less likely to be current smokers than evening people. These comparisons were similar in the subset of individuals (*n* = 130,737) with MHQ data and information on diurnal preference available (Supplementary Table 3) and in unrelated individuals (Supplementary Table 4).

**Table 1:** Demographics and lifestyle characteristics of morning and evening people in all individuals and men only and women only.

	All individuals			Men only			Women only		
	Morning person (cases)	Evening person (controls)	*P*^a^	Morning person (cases)	Evening person (controls)	*P*^a^	Morning person (cases)	Evening person (controls)	*P*^a^
*N*	252,240	150,888		110,419	68,526		141,821	82,362	
*N* Male (%)	110,419 (43.8)	68,526 (45.4)	<1 × 10^−15^						
Mean age (SD)	57.8 (7.8)	56.4 (8.2)	<1 × 10^−15^	58.1 (7.9)	56.4 (8.4)	<1 × 10^−15^	57.6 (7.8)	56.4 (8.1)	<1 × 10^−15^
Mean BMI (SD)	27.3 (4.7)	27.6 (4.9)	<1 × 10^−15^	27.9 (4.2)	27.9 (4.3)	0.32	26.9 (5.0)	27.3 (5.3)	<1 × 10^−15^
Mean TDI (SD)	−1.57 (2.9)	−1.32 (3.1)	2 × 10^−8^	−1.56 (3.0)	−1.25 (3.1)	<1 × 10^−15^	−1.58 (2.9)	−1.38 (3.0)	0.75
Smoking status			<1 × 10^−15^			<1 × 10^−15^			<1 × 10^−15^
Never smoker (%)	141,599 (56.1)	74,301 (49.2)		55,162 (50.0)	30,340 (44.3)		86,437 (60.9)	43,961 (53.4)	
Former smoker (%)	88,783 (35.2)	54,734 (36.3)		43,787 (39.7)	26,907 (39.3)		44,996 (31.7)	27,827 (33.8)	
Current smoker (%)	18,949 (7.5)	19,460 (12.9)		9959 (9.0)	9973 (14.6)		8,990 (6.3)	9,487 (11.5)	
Missing (%)	2,909 (1.2)	2,393 (1.6)		1,511 (1.4)	1306 (1.9)		1,398 (0.99)	1,087 (1.3)	
Depressive symptoms (%)	25,759 (10.2)	18,834 (12.5)	<1 × 10^−15^	8819 (8.0)	6907 (10.1)	<1 × 10^−15^	16,940 (12.0)	11,927 (14.5)	<1 × 10^−15^
Major depression^b^ (%)	18,232 (7.2)	12,876 (8.5)	<1 × 10^−15^	5282 (4.8)	4084 (6.0)	<1 × 10^−15^	12,950 (9.1)	8,792 (10.7)	<1 × 10^−15^
Mean CIDI severity^b^ (SD)	2.88 (2.9)	3.25 (3.0)	<1 × 10^−15^	2.15 (2.7)	2.56 (2.8)	<1 × 10^−15^	3.40 (3.0)	3.77 (3.0)	<1 × 10^−15^
Mean PHQ9 severity^b^ (SD)	2.63 (3.5)	3.13 (4.0)	<1 × 10^−15^	2.30 (3.4)	2.80 (3.9)	<1 × 10^−15^	2.86 (3.6)	3.39 (4.1)	<1 × 10^−15^
Mean wellbeing score^b^ (SD)	12.8 (1.9)	12.4 (2.1)	<1 × 10^−15^	12.8 (2.0)	12.4 (2.0)	<1 × 10^−15^	12.8 (1.9)	12.4 (2.1)	<1 × 10^−15^
GAD^b^ (%)	3,813 (1.5)	2,424 (1.6)	3 × 10^−12^	1260 (1.1)	1009 (1.5)	5 × 10^−6^	2,553 (1.8)	1,815 (2.2)	5 × 10^−8^
Mean GAD severity^b^ (SD)	2.09 (3.3)	2.32 (3.5)	5 × 10^−10^	1.68 (3.0)	1.95 (3.3)	3 × 10^−6^	2.38 (3.5)	2.61 (3.7)	1 × 10^−5^
Composite Phase Deviation, CPD^c^ (SD)	1.06 (0.7)	1.13 (0.8)	<1 × 10^−15^	1.07 (0.7)	1.16 (0.8)	2 × 10^−11^	1.06 (0.7)	1.12 (0.7)	3 × 10^−8^

^a^*P* comparison of morning and evening people using logistic regression adjusted for age, sex (for all individuals), assessment centre, Townsend Deprivation Index (TDI), Body Mass Index (BMI) and smoking status.

^b^Available in a subset of individuals (up to 146,067) from the UK Biobank who participated in the mental health questionnaire.

^c^The actigraphy measure, CPD, was adjusted for age at actigraphy, season of actigraphy, sex (for all individuals), assessment centre Townsend Deprivation Index (TDI), Body Mass Index (BMI) and smoking status.

### Diurnal preference and depression

**Fig. 4:**
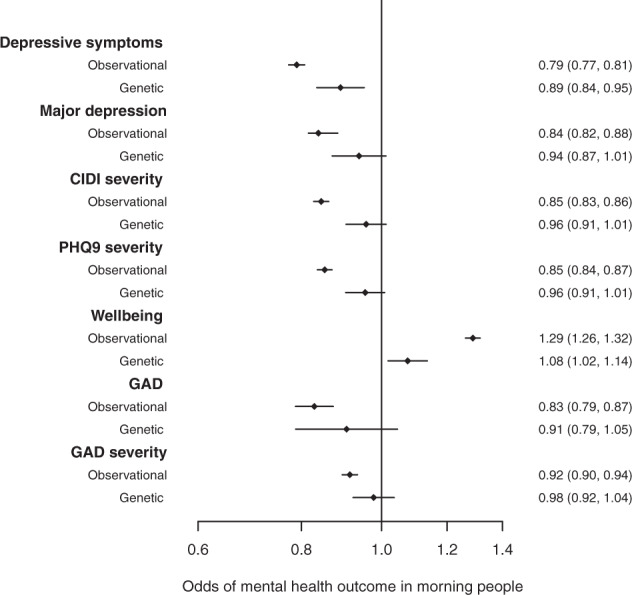
The observational and genetic associations between increased morningness and the odds of the seven mental health and wellbeing outcomes with eveningness as the referent. To be consistent with the observational results, we present the genetic 1-sample ln(OR) rather than the twofold increase as described in the methods.

Observationally, diurnal preference was robustly associated with depression. Individuals reporting to be morning people had lower odds of depressive symptoms (OR: 0.79, 95% CI: 0.77, 0.81) when compared to evening people. Similar results were observed when using the MHQ derived measures. For example, morning people had lower odds of major depression (OR: 0.84, 95% CI: 0.82, 0.86) than evening people (Fig. 4). Results were consistent in males and females (Supplementary Fig. 2a).

MR provided further evidence that morningness was protective for depression. Using one-sample MR, a twofold higher genetic liability of morningness was associated with lower odds of depressive symptoms (OR: 0.92, 95% CI: 0.88, 0.97). The direction of effect was similar using the more detailed MHQ depression measures, although the confidence intervals generally crossed the null (Fig. 4). Results were consistent in males and females (Supplementary Fig. 2a).

Using two-sample MR approaches provided consistent results. For example, using the IVW method, a twofold higher genetic liability towards morningness was associated lower odds of depressive symptoms (OR:0.97, 95% CI: 0.95, 1.00). The more pleiotropy robust methods (MR Egger and Median MR) generally demonstrated consistent results, suggesting minimal horizontal pleiotropy (Supplementary Table 5; Supplementary Fig. 3a). Effect sizes were similar for males and females and were consistent when using the smaller subset of 108 variants (Supplementary Fig. 4). Effect estimates showed directional consistency when using the MHQ derived depression measures, although power was limited. For example, a twofold higher genetic risk of morningness was associated with lower odds of current depression severity (OR:0.95, 95% CI: 0.90, 0.99) measured by the PHQ9.

### Diurnal preference and wellbeing

Observationally, individuals reporting to be morning people had higher wellbeing than evening people. For example, morning people had higher odds of a higher wellbeing score (OR: 1.29, 95% CI:1.26, 1.32; Fig. 4). Using one-sample MR approaches, a twofold higher genetic liability of morningness increased the odds of higher wellbeing by 5% (OR:1.05, 95%CI: 1.01, 1.10; Fig. 4). The effect estimates were similar in sex-stratified analyses although in males, the confidence intervals crossed the null (Supplementary Fig. 2b).

Two-sample MR methods were generally consistent. The IVW method provided evidence that a twofold higher genetic liability towards morningness was associated with higher wellbeing (OR:1.06, 95% CI: 1.03, 1.08). MR-Egger and Median MR were directionally consistent (Supplementary Table 5; Supplementary Fig. 3b) although, the confidence intervals were much wider. The MR-Egger intercept suggested weak evidence of horizontal pleiotropy (*P* = 0.03). The sex-stratified analyses were similar, with a twofold higher genetic liability towards morningness in the IVW model associated with a 13% (OR:1.13) and 11% (OR:1.11) higher wellbeing in males and females, respectively. Using the 23andMe identified SNPs only, the effect estimates were consistent, but confidence intervals crossed null (Supplementary Fig. 4).

### Diurnal preference and anxiety

Although observational analysis suggested evidence for an inverse relationship between morningness and anxiety (OR:0.83, 95% CI: 0.79, 0.87) the confidence intervals, in one-sample MR analysis, were too wide for confident interpretation (OR:0.91, 95% CI: 0.79, 1.05) (Fig. 4). This was similar in (a) the sex-stratified analysis (Supplementary Fig. 2c, b) the two-sample MR analyses (Supplementary Table 5; Supplementary Fig. 3c).

### Sensitivity analysis

#### Shift work

Observationally, stratification of analyses by shift working status demonstrated that morningness was associated with lower odds of depressive symtoms, major depression, depression severity, anxiety and anxiety severity and higher odds of improved wellbeing in shift workers and non-shift workers, similar to all individuals (Supplementary Fig. 5). In one-sample MR analyses, the effect estimates were generally consistent in non-shift workers whilst estimates for shift workers, due to reduced numbers, had very large confidence intervals making interpretation difficult (Supplementary Fig. 5).

#### Sleep disturbance

We removed individuals reporting to have disrupted sleep in the CIDI, PHQ9 and GAD7 questionnaires. Observationally effect estimates were consistent with increased morningness trending with reduced lifetime depression severity, current depression severity, lifetime anxiety and anxiety severity (Supplementary Fig. 6).

### Diurnal preference and behavioural circadian misalignment (CPD)

Observationally, having an early diurnal preference was associated with lower CPD (*β*: −0.07 SD, 95% CI: −0.08 SD, −0.05 SD) i.e. morningness was associated with decreased circadian misalignment (Supplementary Table 6).

One-sample MR suggested that a doubling in the genetic liability for morningness was associated with lower CPD (*β*: −0.02 SD, 95% CI: −0.06 SD, 0.02 SD) representing lower misalignment although, the effect estimates crossed the null (Supplementary Table 6). Two-sample MR methods were generally consistent. The IVW method showed a twofold higher genetic liability towards morningness was associated with lower CPD (*β*: −0.04 SD, 95% CI: −0.07 SD, −0.01 SD). Median MR and MR-Egger showed directional consistency although the effect estimates for MR-Egger crossed the null (Supplementary Table 6).

### Behavioural circadian misalignment and mental health

**Fig. 5:**
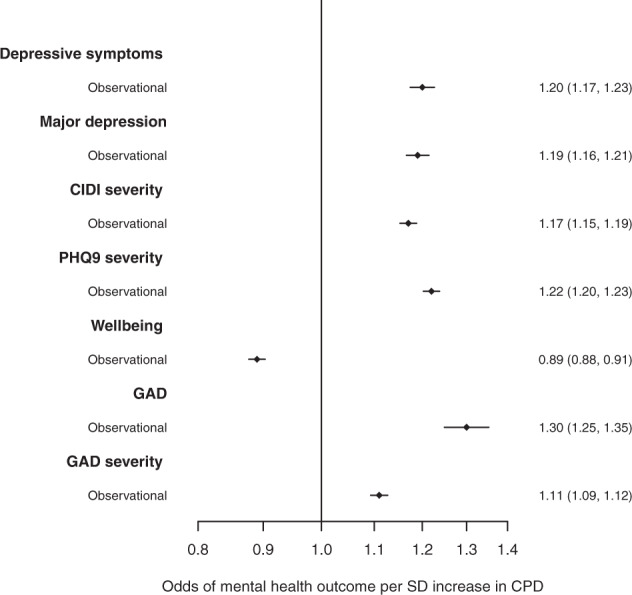
Logistic or ordinal logistic regression models were used to test the observational associations between CPD and the mental health and wellbeing outcomes. Models were adjusted for age at actigraphy, sex and season of actigraphy wear.

Observationally, we demonstrated that increased circadian misalignment, using CPD, was robustly associated with higher odds of depression and anxiety and lower odds of higher wellbeing (Fig. 5). For example, a one standard deviation (SD) higher CPD was associated with higher odds of depressive symptoms (OR: 1.20, 95% CI: 1.17, 1.23), major depression (OR:1.19, 95% CI: 1.16, 1.21) and anxiety (OR:1.30, 95% CI:1.25, 1.35) and lower odds of wellbeing (OR: 0.89, 95%CI: 0.88, 0.91) (Supplementary Table 7).

Several sensitivity analyses were performed to test the validity of these findings. When stratified by sex and age (above and below 65, assuming 65 years as the age of retirement), the results remained consistent with highly misaligned individuals having higher odds of depression and anxiety and lower wellbeing (Supplementary Figs. 7, 8). In addition, when adjusting for diurnal preference and the diurnal preference GRS the results remained consistent (Supplementary Fig. 9). Furthermore, the effect sizes were similar in non-shift workers and shift workers although the confidence intervals were wider in shift workers (Supplementary Fig. 10). Using an alternative measure of CPD that adjusts for sleep-duration-corrected ‘free-day’ (weekend) midsleep timings (MSFsc) results were consistent (Supplementary Fig. 11) [12]. For example, an increase in CPD (using all-day midsleep) was associated with higher odds of depressive symtoms by 20% (OR: 1.20, 95% CI:1.17, 1.23), whilst the CPD adjusted for MSFsc (midsleep on ‘free’ days) was associated with 19% higher odds of depressive symptoms (OR: 1.19, 95% CI: 1.16, 1.22). Finally, stratifying by relevant medication status (medications affecting sleep and mental health; online supplement), resulted in similar findings (Supplementary Fig. 12).

## Discussion

Using genetic variants for diurnal preference, this study adds to the evidence base that being a morning person lowers the likelihood of depressive symptoms and major depression, improves wellbeing, and associates with less circadian misalignment, as assessed by objective, actigraphy measures of sleep variability that serve as a behavioural misalignment proxy. Furthermore, we show that higher misalignment is associated with higher odds of depression and anxiety and poorer wellbeing.

Our findings provide further evidence that morningness improves mental health and wellbeing as suggested in previous observational and genetic studies [6, 10, 43, 44]. A recent paper utilised summary statistic data and two-sample MR methods to demonstrate that morningness was associated with reduced odds of schizophrenia and depressive symptoms and higher odds of subjective wellbeing [10]. Here, our study extends this work by using the MHQ data in UK Biobank to further test these relationships using well-validated mental health and wellbeing outcomes [23]. Our findings were generally consistent with previous studies, providing evidence that the odds of major depression and depression severity were lower in morning people, whilst wellbeing was higher.

The availability of individual-level data in the UK Biobank allowed us to test a number of hypotheses that are challenging to perform using two-sample MR and GWAS consortium summary statistics. This included the stratification by sex and shift worker status. Whilst women were more likely to report a diagnosis of depression in the UK Biobank and there is some evidence for sex-specific effects in chronotype and health associations, the effect estimates obtained here for the role of diurnal preference on mental health and wellbeing were similar in both sexes in observational and genetic models [29, 45]. Previous research suggested that higher depressive symptoms, using the Center for Epidemiologic Studies Depression Scale to define “possible depression”, were seen in shift workers compared to non-shift workers due to a delay in the central circadian clock on shift working days [46]. Additionally, shift workers matching their chronotype to their shift time had improved wellbeing and shift worker disorder was associated with anxiety and higher anxiety severity [47, 48]. Here, we provide some evidence that morningness is not protective for depression and wellbeing in shift workers. However, these associations were uncertain because of low numbers and reduced power, resulting in wide confidence intervals. Furthermore, the lack of findings for the association between diurnal preference and anxiety is in line with previous literature [6]. Observational associations are likely to relate to an individuals interpretation of anxiety-related.

Circadian misalignment is a potential explanation for the link between diurnal preference and mental health and wellbeing, with evening people tending to be more misaligned [13, 49]. The actigraphy data in UK Biobank provided a unique opportunity to quantitatively test the role of misalignment in mental health and wellbeing. We provided evidence that a genetic liability to morningness had a nominal effect on CPD; morning people were more aligned. Observationally, we demonstrated that more misaligned individuals (i.e., higher CPD) were more likely to report depression, anxiety and have lower wellbeing. This was true when several sensitivity analyses were performed including stratification by sex, age, diurnal preference, relevant medication usage and shift worker status. These analyses strengthen the evidence that circadian misalignment has adverse effects on mental health and may partially explain the links between diurnal preference and mental health although reverse causation (depression disrupting sleep patterns and causing misalignment) can not be ruled out and should be tested in future work. Furthermore, future work should aim to use weak instrument MR methods to identify valid genetic instruments for CPD to test the causal role of CPD in mental health and wellbeing.

Our findings fit with previous evidence that evening people may experience more circadian misalignment, as their chronotype is often mismatched with diurnal (9–5) schedules, which are the societal norm [16]. Individuals with a physiological tendency towards delayed sleep and circadian timing are especially prone to further delay by modern schedules and lighting, resulting in greater social jet-lag [50]. Our findings also build on existing evidence of circadian misalignment in shift workers, who often work against their diurnal preference, with some studies suggesting that these individuals have a higher prevalence of depression and lower wellbeing [51, 52].

### Strengths and limitations

The major strength of this study was the availability of well-validated mental health and wellbeing data in 146,067 individuals of whom 61,238 also had actigraphy information available. This ensured sufficient power for MR analyses and allowed us to use a high-resolution measure of behavioural circadian misalignment. However, there were a number of limitations to our approach. First, the UK Biobank is not population representative and therefore findings might not be generalisable to the UK population [21]. However, UK Biobank does appear to provide valid assessments of risk factor associations that appear to be widely generalisable [53]. Second, both the actigraphy and MHQ data were only analysed in a subset of individuals and this may introduce further biases that could influence our observational and MR findings [54, 55]. Third, the diurnal preference variants utilised in MR were discovered using UK Biobank, which has the potential to induce biases into the data, especially “winner’s curse”, which can lead to underestimation of the true causal effects. However, we took approaches to minimise this, by weighting those variants by their effects on diurnal preference excluding UK Biobank and repeating our analyses using the 108 variants that were discovered in 23andMe alone. Similar findings were observed with both approaches. Finally, there was no contemporary information on retirement status and shift working with the actigraphy data and no details of which days of the actigraphy recordings were free days or workdays. The assumption was made that the all-day mean sleep midpoint was representative of an individual’s sleep timing for the primary CPD measure, which may have led to subtle biases between working and retired individuals. Sensitivity analyses, however, were undertaken to try to assess whether these biases were significantly influencing the results and they showed results that were generally consistent.

In conclusion, we have provided evidence that being an early-type (i.e., a morning person) is protective against depression and improves general wellbeing. This may in part be explained by the lower circadian misalignment observed in morning people, however, further work should aim to establish whether the effects of genetic diurnal preference on depression and wellbeing is mediated through circadian misalignment. These analyses are required to fully understand this relationship and if confirmed, could mean that introducing further flexibility to the working day may improve mental health and wellbeing in evening people.

## Supplementary information

Online supplement

## References

[1] Dibner C, Schibler U (2015). Circadian timing of metabolism in animal models and humans. J Intern Med.

[2] Kalmbach DA, Schneider LD, Cheung J, Bertrand SJ, Kariharan T, Pack AI, et al. Genetic basis of chronotype in humans: insights from three landmark GWAS. Sleep. 2017;40:zsw048. 10.1093/sleep/zsw048 PMC6084759 28364486

[3] Konttinen H, Kronholm E, Partonen T, Kanerva N, Mannisto S, Haukkala A (2014). Morningness-eveningness, depressive symptoms, and emotional eating: a population-based study. Chronobiol Int.

[4] Alvaro PK, Roberts RM, Harris JK (2014). The independent relationships between insomnia, depression, subtypes of anxiety, and chronotype during adolescence. Sleep Med.

[5] Merikanto I, Lahti T, Puolijoki H, Vanhala M, Peltonen M, Laatikainen T (2013). Associations of chronotype and sleep with cardiovascular diseases and type 2 diabetes. Chronobiol Int.

[6] Kivela L, Papadopoulos MR, Antypa N (2018). Chronotype and psychiatric disorders. Curr Sleep Med Rep.

[7] Vetter C, Chang S-C, Devore EE, Rohrer F, Okereke OI, Schernhammer ES (2018). Prospective study of chronotype and incident depression among middle- and older-aged women in the Nurses’ Health Study II. J Psychiatr Res.

[8] Willis TA, O’Connor DB, Smith L (2005). The influence of morningness-eveningness on anxiety and cardiovascular responses to stress. Physiol Behav.

[9] Díaz-Morales J, Sánchez-López M (2008). Morningness-eveningness and anxiety among adults: A matter of sex/gender?. Personal Individ Differ.

[10] Jones SE, Lane JM, Wood AR, van Hees VT, Tyrrell J, Beaumont RN (2019). Genome-wide association analyses of chronotype in 697,828 individuals provides insights into circadian rhythms. Nat Commun.

[11] Okbay A, Baselmans BML, De Neve J-E, Turley P, Nivard MG, Fontana MA (2016). Genetic variants associated with subjective well-being, depressive symptoms, and neuroticism identified through genome-wide analyses. Nat Genet.

[12] Fischer D, Vetter C, Roenneberg T (2016). A novel method to visualise and quantify circadian misalignment. Sci Rep.

[13] Potter GD, Skene DJ, Arendt J, Cade JE, Grant PJ, Hardie LJ (2016). Circadian rhythm and sleep disruption: causes, metabolic consequences, and countermeasures. Endocr Rev.

[14] Vetter C (2020). Circadian disruption: what do we actually mean?. Eur J Neurosci.

[15] Roenneberg T, Merrow M (2016). The circadian clock and human health. Curr Biol.

[16] Roenneberg T, Pilz LK, Zerbini G, Winnebeck EC. Chronotype and social jetlag: a (self-) critical review. Biology (Basel). 2019;8:54. 10.3390/biology8030054 PMC6784249 31336976

[17] Wittmann M, Dinich J, Merrow M, Roenneberg T (2006). Social jetlag: misalignment of biological and social time. Chronobiol Int.

[18] Tavernier R, Munroe M, Willoughby T (2015). Perceived morningness-eveningness predicts academic adjustment and substance use across university, but social jetlag is not to blame. Chronobiol Int.

[19] Islam Z, Hu H, Akter S, Kuwahara K, Kochi T, Eguchi M, et al. Social jetlag is associated with an increased likelihood of having depressive symptoms among the Japanese working population: the Furukawa Nutrition and Health Study. Sleep. 2020;43:zsz204. 10.1093/sleep/zsz204 PMC6985924 31555821

[20] Borisenkov MF, Petrova NB, Timonin VD, Fradkova LI, Kolomeichuk SN, Kosova AL (2015). Sleep characteristics, chronotype and winter depression in 10-20-year-olds in northern European Russia. J Sleep Res.

[21] Sudlow C, Gallacher J, Allen N, Beral V, Burton P, Danesh J (2015). UK biobank: an open access resource for identifying the causes of a wide range of complex diseases of middle and old age. PLoS Med.

[22] Bycroft C, Freeman C, Petkova D, Band G, Elliott LT, Sharp K (2018). The UK Biobank resource with deep phenotyping and genomic data. Nature..

[23] Davis KAS, Coleman JRI, Adams M, Allen N, Breen G, Cullen B, et al. Mental health in UK Biobank – development, implementation and results from an online questionnaire completed by 157 366 participants. BJPsych Open. 2020;6:e18. 10.1192/bjo.2019.100 PMC7176892 32026800

[24] Jones SE, van Hees VT, Mazzotti DR, Marques-Vidal P, Sabia S, van der Spek A (2019). Genetic studies of accelerometer-based sleep measures yield new insights into human sleep behaviour. Nat Commun.

[25] Megdal SP, Schernhammer ES (2007). Correlates for poor sleepers in a Los Angeles high school. Sleep Med.

[26] Horne JA, Östberg O (1976). A self-assessment questionnaire to determine morningness-eveningness in human circadian rhythms. Int J Chronobiol.

[27] Kitamura S, Hida A, Aritake S, Higuchi S, Enomoto M, Kato M (2014). Validity of the Japanese version of the Munich ChronoType Questionnaire. Chronobiol Int.

[28] Kantermann T, Sung H, Burgess HJ (2015). Comparing the Morningness-Eveningness Questionnaire and Munich ChronoType Questionnaire to the dim light melatonin onset. J Biol Rhythms.

[29] Tyrrell J, Mulugeta A, Wood AR, Zhou A, Beaumont RN, Tuke MA (2018). Using genetics to understand the causal influence of higher BMI on depression. Int J Epidemiol.

[30] Davis KAS, Coleman JRI, Adams M, Allen N, Breen G, Cullen B (2020). Mental health in UK Biobank - development, implementation and results from an online questionnaire completed by 157 366 participants: a reanalysis. BJPsych Open.

[31] Levis B, Benedetti A, Thombs BD (2019). Accuracy of Patient Health Questionnaire-9 (PHQ-9) for screening to detect major depression: individual participant data meta-analysis. BMJ..

[32] Xiao R, Boehnke M (2009). Quantifying and correcting for the winner’s curse in genetic association studies. Genet Epidemiol.

[33] Davies NM, Holmes MV, Davey Smith G (2018). Reading Mendelian randomisation studies: a guide, glossary, and checklist for clinicians. BMJ.

[34] Loh P-R, Tucker G, Bulik-Sullivan BK, Vilhjálmsson BJ, Finucane HK, Salem RM (2015). Efficient Bayesian mixed-model analysis increases association power in large cohorts. Nat Genet.

[35] Burgess S, Thompson SG (2017). Interpreting findings from Mendelian randomization using the MR-Egger method. Eur J Epidemiol.

[36] Bowden J, Del Greco MF, Minelli C, Davey Smith G, Sheehan NA, Thompson JR (2016). Assessing the suitability of summary data for two-sample Mendelian randomization analyses using MR-Egger regression: the role of the I2 statistic. Int J Epidemiol.

[37] Bowden J, Davey, Smith G, Haycock PC, Burgess S (2016). Consistent estimation in Mendelian randomization with some invalid instruments using a weighted median estimator. Genet Epidemiol.

[38] Brion M-JA, Shakhbazov K, Visscher PM (2013). Calculating statistical power in Mendelian randomization studies. Int J Epidemiol.

[39] Lawlor DA (2016). Commentary: Two-sample Mendelian randomization: opportunities and challenges. Int J Epidemiol.

[40] Burgess S, Labrecque JA (2018). Mendelian randomization with a binary exposure variable: interpretation and presentation of causal estimates. Eur J Epidemiol.

[41] Roenneberg T, Wirz-Justice A, Merrow M (2003). Life between clocks: daily temporal patterns of human chronotypes. J Biol Rhythms.

[42] Juda M, Vetter C, Roenneberg T (2013). The Munich ChronoType Questionnaire for shift-workers (MCTQShift). J Biol Rhythms.

[43] Takahashi JS, Hong HK, Ko CH, McDearmon EL (2008). The genetics of mammalian circadian order and disorder: implications for physiology and disease. Nat Rev Genet.

[44] Toomey R, Panizzon MS, Kremen WS, Franz CE, Lyons MJ (2015). A twin-study of genetic contributions to morningness–eveningness and depression. Chronobiol Int.

[45] Fabbian F, Zucchi B, De Giorgi A, Tiseo R, Boari B, Salmi R (2016). Chronotype, gender and general health. Chronobiol Int.

[46] Togo F, Yoshizaki T, Komatsu T (2017). Association between depressive symptoms and morningness-eveningness, sleep duration and rotating shift work in Japanese nurses. Chronobiol Int.

[47] Vetter C, Fischer D, Matera JL, Roenneberg T (2015). Aligning work and circadian time in shift workers improves sleep and reduces circadian disruption. Curr Biol.

[48] Flo E, Pallesen S, Mageroy N, Moen BE, Gronli J, Hilde Nordhus I (2012). Shift work disorder in nurses–assessment, prevalence and related health problems. PLoS ONE.

[49] Baron KG, Reid KJ (2014). Circadian misalignment and health. Int Rev Psychiatry.

[50] Swaminathan K, Klerman EB, Phillips AJK (2017). Are individual differences in sleep and circadian timing amplified by use of artificial light sources?. J Biol Rhythms.

[51] James SM, Honn KA, Gaddameedhi S, Van Dongen HPA (2017). Shift work: disrupted circadian rhythms and sleep-implications for health and well-being. Curr Sleep Med Rep.

[52] Kalmbach DA, Pillai V, Cheng P, Arnedt JT, Drake CL (2015). Shift work disorder, depression, and anxiety in the transition to rotating shifts: the role of sleep reactivity. Sleep Med.

[53] Batty GD, Gale CR, Kivimäki M, Deary IJ, Bell S (2020). Comparison of risk factor associations in UK Biobank against representative, general population based studies with conventional response rates: prospective cohort study and individual participant meta-analysis. BMJ..

[54] Adams MJ, Hill WD, Howard DM, Dashti HS, Davis KAS, Campbell A, et al. Factors associated with sharing e-mail information and mental health survey participation in large population cohorts. Int J Epidemiol. 2020;49:410–21. 10.1093/ije/dyz134 PMC7266553 31263887

[55] Tyrrell J, Zheng J, Beaumont R, Hinton K, Richardson TG, Wood AR, et al. Genetic predictors of participation in optional components of UK Biobank. 2020. https://www.biorxiv.org/content/10.1101/2020.02.10.941328v1.full. 10.1038/s41467-021-21073-y PMC7873270 33563987

