# Supplementary material for: Using Mendelian Randomisation methods to understand whether diurnal preference is causally related to mental health
Source: Mol Psychiatry. 2021 Jun 8;26(11):6305–16. doi: 10.1038/s41380-021-01157-3 (PMC8760058; doi:10.1038/s41380-021-01157-3)
Supplement: Supplementary file 1 — Online supplement [file 41380_2021_1157_MOESM1_ESM.docx]

**Supplementary Material**

Using Mendelian Randomization methods to understand whether diurnal preference is causally related to mental health

**Methods**

**Exposures**

**Circadian Misalignment**

*Actigraphy data in the UK Biobank*

UK Biobank participants were invited to wear an activity monitor for one week. Those that participated in this sub-study were mailed an Axivity AX3 device (specifications at <https://axivity.com/userguides/ax3/>) and were asked to wear the device on the dominant wrist continuously for seven days, including while sleeping, showering and bathing. A total of 103,720 participants returned the device and had data recorded over at least three complete 24-hour periods. Detailed information on protocol and participant instructions can be found online at <https://biobank.ctsu.ox.ac.uk/crystal/crystal/docs/PhysicalActivityMonitor.pdf> and <https://biobank.ctsu.ox.ac.uk/crystal/crystal/images/activity_invite.png> (UK Biobank resources 131600 and 141141 respectively). The raw actigraphy data files for each individual were downloaded in Continuous Wave Accelerometer (cwa) format (UK Biobank data field 90001) and converted to Waveform Audio File (wav) format using the open-source software OMConvert, recommended by the device’s manufacturer Axivity (see <https://github.com/digitalinteraction/openmovement/tree/master/Software/AX3/omconvert>).

*Identification of sleep periods*

The R package GGIR (1) (2) (v1.6-9) was used to process the converted UK Biobank actigraphy data files (in .wav format) and provide summaries of activity and sleep. GGIR uses a heuristic algorithm (3) to determine sleep (and rest) periods by:

1. Estimating the median z-angle (perpendicular to the wrist) every 5 seconds windows (epochs) using the three perpendicular axes of motion in relation to the downward force of gravity
2. Calculating a 5-minute rolling median of the absolute differences in z-angle between the 5 second epochs
3. Determining the 10th percentile of the rolling median across a day (from noon to noon) and multiply by 15 to set a threshold for determining inactivity
4. Detecting blocks of activity for which the rolling median z-angle difference (from step 2) is less than the threshold (from step 3) and which last for 30 or more minutes
5. Combining blocks of inactivity, determined in step 4, which are separated by less than 60 minutes
6. Labelling the longest combined period of sustained inactivity (from step 5) in each day (noon-noon) as the sleep period time windows (SPT window) and all other periods of sustained inactivity as diurnal (daytime) inactivity.

The threshold used for detecting sustained inactivity means that those detected outside the SPT window can be considered as waking rest or naps (2). The full list of variables produced by GGIR can be found in the CRAN GGIR reference manual (see <https://cran.r-project.org/web/packages/GGIR/GGIR.pdf>).

*Determination of valid nights and sleep midpoint*

GGIR provides both daily/nightly summaries and averages across the whole wear period. Using the nightly data from the “part 4” results file, we determined valid nights to be those with SPT window length (“acc_wake” – “acc_onset”) greater than or equal to 3 hours, less than or equal to 12 hours and with 2 or more sleep episodes (“Nblocks_nightsleep”) during the SPT window, in order to exclude nights potentially containing undetected periods of non-wear. To exclude nights with errors in sleep onset and offset prediction, a custom script was used to identify cases where sustained inactivity did not match predicted sleep-wake state in 1.5h windows before and after sleep onset and offset. Sleep midpoint was then calculated as the time halfway between sleep onset and waking (“acc_wake”/2 + “acc_onset”/2) on valid nights only, with sleep midpoints set to missing on invalid nights.

*Derivation of Compositive Phase Deviation (CPD)*

The Composite Phase Deviation combines the interdaily variability of sleep timing and the variability from each individual’s assumed typical sleep timing. CPD is considered a high-resolution measure of social jetlag and was calculated in two ways, only differing by how the individual’s typical sleep timing was calculated.

To calculate CPD, $\Delta REF$, the difference between each night’s sleep midpoint and the reference sleep midpoint, was first calculated. For the primary CPD measure, the reference sleep midpoint was the mean sleep midpoint across all nights whereas for the secondary CPD measure, used for sensitivity analyses, the mean sleep midpoint on “free” (Friday and Saturday) nights (MSFsc) was utilised, with a correction applied for oversleeping on those nights(4, 5). The difference between each night’s sleep midpoint and the previous night, $\Delta DD$, was then calculated with $\Delta DD$ set to missing for the individual’s first night. The CPD for night $i$ was calculated as the Euclidean norm of $\Delta REF$ and $\Delta DD$:

$$CPD_{i}= \sqrt{\Delta REF_{i}^{2}+\Delta DD_{i}^{2}}$$

The per-individual CPD measure was then simply calculated as the mean across all calculated days for each individual:

$$CPD= \frac{1}{n}\sum_{i=1}^{n} CPD_{i}$$

In the original definition of CPD(5), sleep duration-corrected free-night mean sleep midpoint (MSFsc) was used as the reference sleep timing, as this was more representative of unrestricted sleep timing. Although some contemporaneous employment information is available in the Employment history online follow-up (UK Biobank category 130), it is only available for around half of the actigraphy subgroup. Given the inability to differentiate between “restricted” and “unrestricted” nights of sleep, the assumption was made that “free nights” were Friday and Saturday night for all individuals. With the “free night” assumption in mind, it was decided that the primary CPD measure would use an all-night mean sleep midpoint and the secondary sensitivity measure would use MSFsc.

*CPD phenotype preparation and exclusions*

Several exclusion criteria were applied to the CPD phenotype prior to observational and genetic analyses. Firstly, individuals with poor quality actigraphy data were excluded from analyses. Samples were considered poor quality if they had any of the following:

- a non-zero or missing value in for “Data problem indicator” (UK Biobank data field 90002)
- a value of 0 (“No”) for the “good wear time” flag (field 90015)
- a value of 0 (“No”) for the “good calibration” flag (field 90016)
- a value of 0 (“No”) for the “calibrated on own data” flag (field 90017)
- a value of > 788 (Q3+1.5IQR) for “data recording errors” (field 90182)
- a non-zero count of “interrupted recording periods” (field 90180).

Secondly, non-European individuals were excluded from the phenotype. White Europeans were defined as those that clustered with 1000 Genomes European samples when adopting a k-means clustering approach to cluster individuals by their genetic principal component values, using the first four principal components. These individuals also had to self-report as “British”, “Irish” or “Any other white background” (values 1001, 1002 and 1003, respectively, in data field 21000).

Thirdly, individuals were excluded if they had less than four weeknight sleep midpoints (Sunday through Thursday) or less than one weekend night (Friday and Saturday) sleep midpoint.

After the above exclusions, the CPD phenotype was then standardised to have a mean of 0 and standard deviation of 1. In all sex-stratified analyses, CPD was first separated by sex before being standardised within each sex. After exclusions, there were 76,334 individuals with a valid CPD phenotype.

**Outcomes**

The mental health questionnaire has detailed information on participants mental health and wellbeing. Below we have provided detailed information on how these phenotypes were defined.

*Major Depression*

Firstly, a severity variable ‘CIDI severity’ was derived using 8 variables from the Composite International Diagnostic Interview (CIDI) score. In each case (unless specifically stated) the options were Yes (score of 1) or No (score of 0):

- Have you ever had a time in your life when you felt sad, blue, or depressed for two weeks or more in a row? (data field 20446)
- Have you ever had a time in your life lasting two weeks or more when you lost interest in most things like hobbies, work, or activities that usually give you pleasure? (data field 20441)
- Did you feel more tired out or low on energy than is usual for you? (data field 20449)
- Did you gain or lose weight without trying, or did you stay about the same weight? (data field 20536). Here any response other than stayed the same, resulted in adding one to the overall CIDI response variable.
- Did your sleep change? (data field 20532)
- Was that: [re sleep change] Waking too early? (data field 20535)
- Did you have a lot more trouble concentrating than usual? (data field 20435)
- Did you think a lot about death - either your own, someone else's or death in general? (data field 20437)

This score represents the severity of depression and was utilised to create a binary major depression variable, where cases were defined based on the following criteria:

- Responded yes to the question: "Have you ever had a time in your life when you felt sad, blue, or depressed for two weeks or more in a row?" (data field 20446)

AND

- Scored >4 in our derived CIDI response variable.

Major depression controls were defined based on the following criteria:

- Responded no to the question: "Have you ever had a time in your life when you felt sad, blue, or depressed for two weeks or more in a row?"

AND

- No self-reported depression and anxiety

AND

- No record of depression in hospital episode data

AND

- No known use of antidepressants

*Current severity of depression*

First, the number of PHQ9 questions where participants had rated that they experienced symptoms on more than half the days were calculated and a severity score was calculated. Using the 9 questions two scores were created: the PHQ9 Items and the PHQ9 Severity. For the PHQ9 Items responding nearly every day or more than half the days scored 1 (unless otherwise stated), whilst the severity score assigned was dependent on the participants response, with not at all, several days, more than half the days and nearly every day scoring 0, 1, 2 and 3 respectively. Each participant was asked “Over the last 2 weeks, how often have you been bothered by any of the following problems”:

- Little interest or pleasure in doing things (data field 20514)
- Feeling bad about yourself or that you are a failure or have let yourself or your family down (data field 20507)
- Feeling down, depressed, or hopeless (data field 20510)
- Trouble concentrating on things, such as reading the newspaper or watching television (data field 20508)
- Trouble falling or staying asleep, or sleeping too much (data field 20517)
- Moving or speaking so slowly that other people could have noticed? Or the opposite - being so fidgety or restless that you have been moving around a lot more than usual (data field 20518)
- Feeling tired or having little energy (data field 20519)
- Thoughts that you would be better off dead or of hurting yourself in some way (data field 20513; note for PHQ9 items this scored one if anything other than not at all was selected)
- Poor appetite or overeating (data field 20511)(6)

*Generalised anxiety disorder (GAD)*

To define GAD a count of somatic anxiety symptoms was created. Participants answering yes to the question “Have you ever had a period lasting one month or longer when most of the time you felt worried, tense, or anxious?” (data field 20421) were then asked “When you were worried or anxious were you also”:

- Restless (data field 20426)
- Keyed up or on edge (data field 20423)
- Easily tired (data field 20429)
- Having difficulty keeping your mind on what you were doing (data field 20419)
- More irritable than usual (data field 20422)
- Having tense, sore, or aching muscles (data field 20417)
- Often having trouble falling or staying asleep (data field 20427)

These were summed to give a score from 1 to 7, where a response of “Yes” to the question scored 1, whilst a response of “No” scored 0.

GAD was then defined if participants met all the following criteria:

1. They had experienced a period of one month or longer where most of the time they felt worried, tense or anxious (data field 20421);
2. They reported the longest period spent worried or anxious as over 6 (data field 20420);
3. They reported that they worried on most days during the worst period of anxiety (data field 20538);
4. They reported they worried a lot more than most people would in a particular situation (data fields 20425 and 20542);
5. They reported that they worried about more than one thing when they felt anxious (data fields 20540 and 20543);
6. They reported that they found it difficult to stop worrying when anxious (data fields 20537, 20539, 20541);
7. They scored at least 3 on the symptom count derived above.

Following these criteria, we defined 7,244 cases with valid genetic data available. Controls were defined as individuals who had completed the mental health questionnaire, who did not match the criteria above and who had scored <5 on the GAD-7 questionnaire (see below). This resulted in 89,665 controls.

*Current GAD*

Current generalised anxiety disorder was assessed using the GAD-7 questionnaire (7). All participants in the mental health questionnaire were asked “Over the last 2 weeks how often have you been bothered by any of the following problems?”:

- Feeling nervous, anxious or on edge (data field 20506)
- Not being able to stop or control worrying (data field 20509)
- Worrying too much about different things (data field 20520)
- Trouble relaxing (data field 20515)
- Being so restless that it is hard to sit still (data field 20516)
- Becoming easily annoyed or irritable (data field 20505)
- Feeling afraid as if something awful might happen (data field 20512)

With the option to respond “Not at all” (scored 0), “Several days” (scored 1), “More than half the days” (scored 2), “Nearly every day” (scored 3). Participant responses were summed. This was used to derive a continuous current GAD severity score ‘GAD severity’.

*Relevant self-report medications*

We excluded any individuals reporting one or more of the following medications (field 20003) at baseline. These include drug ingredients and brand names:

Sleep medications: oxazepam, meprobamate, medazepam, bromazepam, lorazepam, clobazam, chlormezanone, temazepam, nitrazepam, lormetazepam, diazepam, zopiclone, triclofos, methyprylone, prazepam, triazolam, ketazolam, dichloralphenazone, clomethiazole, zaleplon, butobarbital, diphenhydramine product, nytol, sonata

Antidepressants: amitriptyline, citalopram, fluoxetine, sertraline, venlafaxine, dosulepin, paroxetine, mirtazapine, escitalopram, trazodone, prozac, seroxat, cipralex, duloxetine, lofepramine, clomipramine, nortriptyline, imipramine, dothiepin, cipramil, amitriptyline, prothiaden, trimipramine, lustral, reboxetine, zispin, cymbalta, anafranil, doxepin, moclobemide, phenelzine, fluvoxamine, yentreve, triptafen, surmontil, tranylcypromine, allegron, edronax, molipaxin, mianserin, nardil, faverin, nefazodone, amitriptyline+chlordiazepoxide, isocarboxazid, manerix, maoi, sinequan, tranylcypromine+trifluoperazine, ludiomil, norval, tryptizol, fluphenazine hydrochloride+nortriptyline.

Antipsychotics: prochlorperazine, olanzapine, quetiapine, risperidone, chlorpromazine, trifluoperazine, amisulpride, sulpiride, seroquel, haloperidol, aripiprazole, stelazine, depixol, flupentixol, clozapine, promazine, risperdal, modecate, fluanxol, flupenthixol, zyprexa, zuclopenthixol, clopixol, largactil, abilify, fluphenazine, haldol, serenace, clozaril, cpz, perphenazine, levomepromazine, pericyazine, dolmatil, fentazin, fluphenazine, benperidol, pimozide, zaponex, denzapine, neulactil, thioridazine, dozic, fluspirilene, panadeine, sertindole.

Anxiolytics: zopiclone, diazepam, temazepam, zolpidem, nitrazepam, lorazepam, hydroxyzine, zimovane, phenergan, promethazine, buspirone, atarax, oxazepam, loprazolam, chlordiazepoxide, lormetazepam, ucerax, stilnoct, diazepam, buspar, alprazolam, librium, xanax, meprate, dalmane, clomethiazole, meprobamate, welldorm, amitriptyline+chlordiazepoxide, flurazepam, heminevrin, medazepam, neulactil, sinequan, almazine, atensine, carisoma, chloractil, chloral, dichloralphenazone, dormonoct, methyprylone, mogadon, rohypnol, tryptizol.

**Supplementary table 1: Lead variants from the METAL meta-analysis of UK Biobank diurnal preference and 23andMe morning person GWAS adapted from Jones et al (2019).**

| **Locus Number** | **Lead Variant** | **Chr** | **Chr Position** | **Morning person allele** | **Non-morning person allele** | **Gene context** | **23andMe Beta** | **SE** |
| --- | --- | --- | --- | --- | --- | --- | --- | --- |
|  |  |  |  |  |  |  |  |  |
| 1 | rs909757 | 1 | 4850823 | T | C | AJAP1-[] | 0.0203 | 0.0062 |
| 2 | rs61773390 | 1 | 7884525 | T | G | [PER3] | 0.0765 | 0.0076 |
| 3 | rs12065331 | 1 | 14507831 | C | T | PRDM2---[]---KAZN | 0.0301 | 0.0066 |
| 4 | rs17448682 | 1 | 15966713 | T | C | [DDI2] | 0.0349 | 0.0071 |
| 5 | rs10917513 | 1 | 20006887 | C | T | HTR6[]-TMCO4 | 0.0345 | 0.0062 |
| 6 | rs10916892 | 1 | 21201325 | C | T | [EIF4G3] | 0.0365 | 0.0061 |
| 7 | rs2506089 | 1 | 24321935 | T | G | SRSF10--[]--MYOM3 | 0.0281 | 0.0064 |
| 8 | rs12140153 | 1 | 62579891 | G | T | [INADL] | 0.0675 | 0.0119 |
| 9 | rs11208844 | 1 | 66851147 | G | A | PDE4B--[]---SGIP1 | 0.0315 | 0.0084 |
| 10 | rs12040629 | 1 | 77705365 | A | G | PIGK--[]--AK5 | 0.0835 | 0.0081 |
| 11 | rs11588913 | 1 | 79963816 | G | A | ELTD1---[] | 0.0208 | 0.0060 |
| 12 | rs5016898 | 1 | 81672013 | C | T | []---LPHN2 | 0.0291 | 0.0060 |
| 13 | rs72720396 | 1 | 91191582 | G | A | BARHL2-[]---ZNF644 | 0.0367 | 0.0073 |
| 14 | rs481214 | 1 | 93469865 | A | T | FAM69A--[]--MTF2 | 0.0202 | 0.0063 |
| 15 | rs11165655 | 1 | 96959104 | G | A | []---PTBP2 | 0.0286 | 0.0060 |
| 16 | rs17575798 | 1 | 110086451 | G | A | [GPR61] | 0.0337 | 0.0076 |
| 17 | rs6690292 | 1 | 113188419 | C | T | [CAPZA1] | 0.0252 | 0.0067 |
| 18 | rs11102807 | 1 | 115061584 | G | A | TRIM33-[]--BCAS2 | 0.0187 | 0.0060 |
| 19 | rs9436119 | 1 | 150467753 | A | G | [TARS2] | 0.0525 | 0.0063 |
| 20 | rs6665637 | 1 | 153756083 | G | A | SLC27A3-[]--GATAD2B | 0.0266 | 0.0069 |
| 21 | rs115073088 | 1 | 174215858 | G | A | [RABGAP1L] | 0.0767 | 0.0183 |
| 22 | rs975025 | 1 | 179338327 | C | T | [AXDND1] | 0.0515 | 0.0114 |
| 23 | rs1144566 | 1 | 182569626 | T | C | [RGS16] | 0.3121 | 0.0184 |
| 24 | rs146820337 | 1 | 190095126 | I | D | [FAM5C] | 0.0398 | 0.0059 |
| 25 | rs1221502 | 1 | 193276975 | A | C | CDC73--[] | 0.0227 | 0.0068 |
| 26 | rs4657983 | 1 | 195454557 | G | A | []---KCNT2 | 0.0309 | 0.0063 |
| 27 | rs6429233 | 1 | 241137033 | A | G | [RGS7] | 0.0217 | 0.0060 |
| 28 | rs13011556 | 2 | 4651923 | G | C | ALLC---[] | 0.0248 | 0.0072 |
| 29 | rs62124718 | 2 | 12822995 | G | A | LPIN1---[]--TRIB2 | 0.0569 | 0.0099 |
| 30 | rs72796401 | 2 | 24180078 | A | T | [UBXN2A] | 0.0288 | 0.0075 |
| 31 | rs6718511 | 2 | 25122324 | A | G | [ADCY3] | 0.0257 | 0.0061 |
| 32 | rs11678584 | 2 | 32563426 | T | A | YIPF4--[]--BIRC6 | 0.0408 | 0.0087 |
| 33 | rs848552 | 2 | 36700580 | G | C | [CRIM1] | 0.0326 | 0.0060 |
| 34 | rs7602499 | 2 | 41385367 | T | C | SLC8A1---[]---C2orf91 | 0.0260 | 0.0064 |
| 35 | rs75120545 | 2 | 44271496 | T | C | LRPPRC--[]---PPM1B | 0.0847 | 0.0181 |
| 36 | rs6544906 | 2 | 46863872 | A | C | CRIPT--[]--SOCS5 | 0.0299 | 0.0060 |
| 37 | rs17396357 | 2 | 48252311 | T | C | FBXO11---[]---FOXN2 | 0.0320 | 0.0061 |
| 38 | rs12470914 | 2 | 50532840 | A | T | [NRXN1] | 0.0687 | 0.0101 |
| 39 | rs4672458 | 2 | 53736362 | C | T | []---ASB3 | 0.0181 | 0.0060 |
| 40 | rs13414393 | 2 | 54275162 | C | T | PSME4--[]--ACYP2 | 0.0213 | 0.0060 |
| 41 | rs10175975 | 2 | 59429807 | T | C | FANCL---[] | 0.0161 | 0.0076 |
| 42 | rs359248 | 2 | 60477461 | G | T | []---BCL11A | 0.0377 | 0.0060 |
| 43 | rs812925 | 2 | 61680993 | G | C | [USP34] | 0.0311 | 0.0062 |
| 44 | rs113851554 | 2 | 66750564 | G | T | [MEIS1] | 0.0673 | 0.0142 |
| 45 | rs2706762 | 2 | 70488470 | C | T | [PCYOX1] | 0.0449 | 0.0083 |
| 46 | rs12464387 | 2 | 75445544 | G | A | TACR1--[]---EVA1A | 0.0236 | 0.0060 |
| 47 | rs6727752 | 2 | 76361783 | A | G | GCFC2---[]---LRRTM4 | 0.0231 | 0.0066 |
| 48 | rs10520176 | 2 | 77217310 | T | C | [LRRTM4] | 0.0386 | 0.0061 |
| 49 | rs11681299 | 2 | 88901732 | T | C | [EIF2AK3] | 0.0353 | 0.0066 |
| 50 | rs34509802 | 2 | 101591710 | A | G | [NPAS2] | 0.0506 | 0.0080 |
| 51 | rs76064513 | 2 | 125438641 | T | C | [CNTNAP5] | 0.0417 | 0.0090 |
| 52 | rs77248969 | 2 | 136490731 | G | A | R3HDM1-[]-UBXN4 | 0.0336 | 0.0087 |
| 53 | rs28380327 | 2 | 144232491 | A | T | [ARHGAP15] | 0.0515 | 0.0062 |
| 54 | rs2166559 | 2 | 149551658 | C | T | EPC2-[]--KIF5C | 0.0253 | 0.0088 |
| 55 | rs747003 | 2 | 161916409 | T | C | RBMS1---[]--TANK | 0.0174 | 0.0061 |
| 56 | rs13004345 | 2 | 174037347 | C | T | [ZAK] | 0.0255 | 0.0062 |
| 57 | rs6433478 | 2 | 175241482 | C | T | [CIR1] | 0.0353 | 0.0061 |
| 58 | rs4666682 | 2 | 186203743 | G | A | ZNF804A---[]---FSIP2 | 0.0290 | 0.0077 |
| 59 | rs11677484 | 2 | 191578172 | T | G | B1--[]---GLS | 0.0175 | 0.0068 |
| 60 | rs1064213 | 2 | 198950240 | A | G | [PLCL1] | 0.0647 | 0.0060 |
| 61 | rs184033703 | 2 | 206956138 | A | G | INO80D-[]--NDUFS1 | 0.0539 | 0.0126 |
| 62 | rs80271258 | 2 | 239311505 | C | T | TRAF3IP1-[]--ASB1 | 0.0962 | 0.0110 |
| 63 | rs62182135 | 2 | 240267305 | C | A | [HDAC4] | 0.0258 | 0.0064 |
| 64 | rs35346733 | 3 | 2521322 | G | A | [CNTN4] | 0.0316 | 0.0076 |
| 65 | rs111261826 | 3 | 7189617 | C | A | [GRM7] | 0.0308 | 0.0064 |
| 66 | rs149611468 | 3 | 8817423 | T | C | OXTR-[]---RAD18 | 0.1739 | 0.0320 |
| 67 | rs6794796 | 3 | 14383632 | A | G | LSM3---[]--SLC6A6 | 0.0209 | 0.0065 |
| 68 | rs9817910 | 3 | 18246870 | G | A | [LOC339862] | 0.0164 | 0.0060 |
| 69 | rs73050286 | 3 | 23224684 | T | C | []--UBE2E2 | 0.0299 | 0.0072 |
| 70 | rs2362775 | 3 | 24924421 | C | T | THRB---[]---RARB | 0.0076 | 0.0060 |
| 71 | rs114848860 | 3 | 36859494 | T | A | DCLK3--[]-TRANK1 | 0.0951 | 0.0207 |
| 72 | rs78580841 | 3 | 46986452 | T | C | [CCDC12] | 0.0456 | 0.0123 |
| 73 | rs12636669 | 3 | 50003323 | T | C | [RBM6] | 0.0708 | 0.0111 |
| 74 | rs17007397 | 3 | 70594975 | C | G | MITF---[]---FOXP1 | 0.0257 | 0.0060 |
| 75 | rs7626335 | 3 | 71575177 | C | A | FOXP1---[]---EIF4E3 | 0.0411 | 0.0064 |
| 76 | rs7429614 | 3 | 77205438 | T | G | [ROBO2] | 0.0460 | 0.0061 |
| 77 | rs112201801 | 3 | 82591379 | C | T | GBE1---[] | 0.0852 | 0.0126 |
| 78 | rs12631477 | 3 | 83804561 | T | C | [] | 0.0279 | 0.0074 |
| 79 | rs1449403 | 3 | 85591467 | A | G | [CADM2] | 0.0545 | 0.0092 |
| 80 | rs34967119 | 3 | 104778430 | A | G | []---ALCAM | 0.0229 | 0.0060 |
| 81 | rs1398346 | 3 | 110271943 | T | C | []---LOC151760 | 0.0325 | 0.0086 |
| 82 | rs1800828 | 3 | 113891549 | C | G | [DRD3] | 0.0158 | 0.0069 |
| 83 | rs72950188 | 3 | 116103275 | T | C | [LSAMP] | 0.0535 | 0.0111 |
| 84 | rs72966564 | 3 | 123149816 | C | T | [ADCY5] | 0.0195 | 0.0073 |
| 85 | rs13065394 | 3 | 132971327 | G | T | [TMEM108] | 0.0239 | 0.0066 |
| 86 | rs4550782 | 3 | 138132393 | T | G | MRAS-[]--ESYT3 | 0.0289 | 0.0063 |
| 87 | rs7649164 | 3 | 150788032 | T | G | CLRN1--[]--MED12L | 0.0242 | 0.0063 |
| 88 | rs6440833 | 3 | 152646244 | A | G | P2RY1--[]---RAP2B | 0.0289 | 0.0060 |
| 89 | rs111867612 | 3 | 157721819 | C | A | C3orf55---[]--SHOX2 | 0.0328 | 0.0102 |
| 90 | rs1599374 | 3 | 160891727 | A | G | B3GALNT1--[]--NMD3 | 0.0372 | 0.0060 |
| 91 | rs3850174 | 3 | 172364093 | T | A | [NCEH1] | 0.0386 | 0.0069 |
| 92 | rs301218 | 3 | 176096919 | G | A | ALADL2---[]---TBL1XR1 | 0.0323 | 0.0061 |
| 93 | rs9836621 | 3 | 182096311 | C | T | SOX2---[]---ATP11B | 0.0403 | 0.0060 |
| 94 | rs1468945 | 3 | 185990392 | G | A | [DGKG] | 0.0355 | 0.0072 |
| 95 | rs3796618 | 4 | 1349602 | T | A | [UVSSA] | 0.0201 | 0.0060 |
| 96 | rs4690085 | 4 | 2697300 | G | A | [FAM193A] | 0.0147 | 0.0060 |
| 97 | rs4698678 | 4 | 18260776 | C | G | LCORL---[] | 0.0378 | 0.0068 |
| 98 | rs1502249 | 4 | 27495379 | A | G | STIM2---[] | 0.0217 | 0.0060 |
| 99 | rs6838677 | 4 | 66520667 | C | A | [EPHA5] | 0.0222 | 0.0064 |
| 100 | rs4860734 | 4 | 67096904 | A | G | EPHA5---[] | 0.0244 | 0.0066 |
| 101 | rs6816922 | 4 | 80206272 | C | A | PAQR3---[]--NAA11 | 0.0223 | 0.0060 |
| 102 | rs6846730 | 4 | 83279041 | C | T | [HNRNPD] | 0.0289 | 0.0070 |
| 103 | rs2850979 | 4 | 102094764 | C | T | [PPP3CA] | 0.0222 | 0.0070 |
| 104 | rs10610420 | 4 | 105317995 | D | I | TACR3---[]--CXXC4 | 0.0394 | 0.0066 |
| 105 | rs7700110 | 4 | 114439894 | A | G | [CAMK2D] | 0.0305 | 0.0068 |
| 106 | rs17455138 | 4 | 130903511 | T | C | C4orf33---[] | 0.0409 | 0.0071 |
| 107 | rs9991917 | 4 | 132512118 | A | T | [] | 0.0453 | 0.0076 |
| 108 | rs4241964 | 4 | 137053959 | G | T | [] | 0.0226 | 0.0060 |
| 109 | rs938836 | 4 | 139939653 | G | A | [CCRN4L] | 0.0267 | 0.0061 |
| 110 | rs72729847 | 4 | 147296930 | C | T | [SLC10A7] | 0.0357 | 0.0074 |
| 111 | rs9997394 | 4 | 163704083 | G | A | FSTL5---[]---NAF1 | 0.0314 | 0.0066 |
| 112 | rs10058356 | 5 | 35220404 | C | T | [PRLR] | 0.0177 | 0.0065 |
| 113 | rs67169439 | 5 | 59027048 | I | D | [PDE4D] | 0.0353 | 0.0061 |
| 114 | rs7701529 | 5 | 63861475 | T | A | [RGS7BP] | 0.0291 | 0.0070 |
| 115 | rs7721608 | 5 | 76581258 | T | G | [PDE8B] | 0.0301 | 0.0060 |
| 116 | rs66507804 | 5 | 86630284 | C | T | [RASA1] | 0.0378 | 0.0075 |
| 117 | rs4269995 | 5 | 87701223 | C | T | TMEM161B---[]---MEF2C | 0.0469 | 0.0069 |
| 118 | rs77960 | 5 | 103964585 | A | G | [] | 0.0161 | 0.0063 |
| 119 | rs1559253 | 5 | 106657015 | A | G | []--EFNA5 | 0.0359 | 0.0063 |
| 120 | rs17140201 | 5 | 115939896 | G | A | SEMA6A--[] | 0.0237 | 0.0080 |
| 121 | rs13172141 | 5 | 122990902 | A | T | CSNK1G3--[]---ZNF608 | 0.0276 | 0.0060 |
| 122 | rs67988891 | 5 | 152204741 | G | C | NMUR2---[]---GRIA1 | 0.0310 | 0.0064 |
| 123 | rs2901796 | 5 | 163330708 | A | G | MAT2B---[] | 0.0258 | 0.0061 |
| 124 | rs42210 | 5 | 166408788 | G | C | []---TENM2 | 0.0309 | 0.0066 |
| 125 | rs12518401 | 5 | 173539588 | G | A | HMP19-[]---MSX2 | 0.0337 | 0.0065 |
| 126 | rs7735794 | 5 | 175339984 | A | G | CPLX2--[]--THOC3 | - | - |
| 127 | rs465670 | 5 | 176877624 | T | C | [PRR7] | 0.0227 | 0.0060 |
| 128 | rs9394154 | 6 | 11574374 | G | C | [TMEM170B] | 0.0189 | 0.0060 |
| 129 | rs34125199 | 6 | 12155114 | D | I | [HIVEP1] | 0.0371 | 0.0062 |
| 130 | rs9381812 | 6 | 13183998 | G | A | [PHACTR1] | 0.0700 | 0.0065 |
| 131 | rs1811899 | 6 | 14878060 | C | T | CD83---[]---JARID2 | 0.0372 | 0.0072 |
| 132 | rs9465253 | 6 | 19102247 | T | C | RNF144B---[]---ID4 | 0.0261 | 0.0066 |
| 133 | rs766406 | 6 | 26319588 | G | T | HIST1H4H--[]--BTN3A2 | 0.0343 | 0.0063 |
| 134 | rs486416 | 6 | 31856070 | G | A | [EHMT2] | 0.0202 | 0.0065 |
| 135 | rs13203140 | 6 | 37630133 | C | T | [MDGA1] | 0.0291 | 0.0062 |
| 136 | rs3923809 | 6 | 38440970 | G | A | [BTBD9] | 0.0291 | 0.0064 |
| 137 | rs12206814 | 6 | 41517457 | C | G | [FOXP4] | 0.0171 | 0.0067 |
| 138 | rs2396004 | 6 | 43355851 | A | G | ZNF318--[]--ABCC10 | 0.0253 | 0.0060 |
| 139 | rs3857599 | 6 | 50938247 | A | C | TFAP2B---[]---PKHD1 | 0.0190 | 0.0081 |
| 140 | rs2653349 | 6 | 55142337 | A | G | [HCRTR2] | 0.0738 | 0.0075 |
| 141 | rs9476310 | 6 | 57767576 | T | C | PRIM2---[]---GUSBP4 | 0.0321 | 0.0060 |
| 142 | rs1931814 | 6 | 62589167 | A | G | [KHDRBS2] | 0.0311 | 0.0060 |
| 143 | rs2881955 | 6 | 72479263 | T | C | OGFRL1---[]---RIMS1 | 0.0276 | 0.0066 |
| 144 | rs12195792 | 6 | 98705295 | A | T | MMS22L---[]---POU3F2 | 0.0471 | 0.0067 |
| 145 | rs11154718 | 6 | 99592404 | C | T | FBXL4---[]---FAXC | 0.0274 | 0.0060 |
| 146 | rs60616179 | 6 | 110244765 | A | G | FIG4--[]--GPR6 | 0.0312 | 0.0132 |
| 147 | rs4535583 | 6 | 115699280 | T | C | []---FRK | 0.0271 | 0.0066 |
| 148 | rs9496623 | 6 | 143751625 | G | A | [ADAT2] | 0.0234 | 0.0068 |
| 149 | rs2050185 | 6 | 147936781 | A | G | SAMD5--[]---SASH1 | 0.0184 | 0.0061 |
| 150 | rs9479402 | 6 | 153135339 | C | T | VIP--[]---FBXO5 | 0.2719 | 0.0270 |
| 151 | rs9347926 | 6 | 165195547 | A | T | []---C6orf118 | 0.0316 | 0.0060 |
| 152 | rs9348050 | 6 | 166263488 | T | C | PDE10A---[]--LINC00473 | 0.0189 | 0.0059 |
| 153 | rs4027217 | 7 | 14093914 | C | A | ETV1--[]--DGKB | 0.0238 | 0.0072 |
| 154 | rs10237162 | 7 | 24085405 | T | C | STK31---[]---NPY | 0.0495 | 0.0066 |
| 155 | rs10951325 | 7 | 32265545 | T | C | [PDE1C] | 0.0370 | 0.0062 |
| 156 | rs6967481 | 7 | 50642701 | T | C | DDC--[]--GRB10 | 0.0359 | 0.0060 |
| 157 | rs4236237 | 7 | 69936477 | C | A | [AUTS2] | 0.0308 | 0.0061 |
| 158 | rs2944831 | 7 | 71779635 | A | G | [CALN1] | 0.0247 | 0.0066 |
| 159 | rs3807651 | 7 | 77823771 | A | T | [MAGI2] | 0.0220 | 0.0061 |
| 160 | rs10254050 | 7 | 96468077 | G | C | SHFM1---[]---DLX6 | 0.0761 | 0.0075 |
| 161 | rs4729854 | 7 | 102383663 | T | A | AK301666--[]-FAM185A | 0.0560 | 0.0069 |
| 162 | rs2396719 | 7 | 113893884 | A | G | PPP1R3A---[]---FOXP2 | 0.0379 | 0.0072 |
| 163 | rs17302081 | 7 | 115673079 | T | C | TFEC-[]---TES | 0.0140 | 0.0060 |
| 164 | rs6968240 | 7 | 121942674 | A | C | [FEZF1] | 0.0331 | 0.0062 |
| 165 | rs62465218 | 7 | 132294312 | C | A | PLXNA4--[]---CHCHD3 | 0.0394 | 0.0086 |
| 166 | rs6958557 | 7 | 133585794 | T | G | [EXOC4] | 0.0350 | 0.0061 |
| 167 | rs113161209 | 7 | 148564367 | A | G | [EZH2] | 0.0393 | 0.0112 |
| 168 | rs2072413 | 7 | 150647969 | C | T | [KCNH2] | 0.0241 | 0.0072 |
| 169 | rs62479736 | 8 | 3654320 | T | G | [CSMD1] | 0.0256 | 0.0066 |
| 170 | rs35524253 | 8 | 4823608 | A | G | [CSMD1] | 0.0468 | 0.0063 |
| 171 | rs2979139 | 8 | 8268313 | G | A | SGK223--[]---CLDN23 | 0.0299 | 0.0060 |
| 172 | rs2322605 | 8 | 27164449 | G | A | [TRIM35] | 0.0250 | 0.0060 |
| 173 | rs71523448 | 8 | 31817493 | G | C | WRN---[]---NRG1 | 0.0627 | 0.0116 |
| 174 | rs6993892 | 8 | 33729200 | C | T | DUSP26---[] | 0.0374 | 0.0062 |
| 175 | rs6468316 | 8 | 35237788 | C | T | [UNC5D] | 0.0147 | 0.0060 |
| 176 | rs7845620 | 8 | 53129069 | C | A | [ST18] | 0.0578 | 0.0080 |
| 177 | rs10109566 | 8 | 59800446 | G | A | [TOX] | 0.0218 | 0.0060 |
| 178 | rs34054660 | 8 | 65015659 | A | G | YTHDF3---[]---BHLHE22 | 0.0332 | 0.0060 |
| 179 | rs187028 | 8 | 73459513 | T | A | [KCNB2] | 0.0276 | 0.0065 |
| 180 | rs16939162 | 8 | 76653156 | A | G | HNF4G---[]---ZFHX4 | 0.0326 | 0.0079 |
| 181 | rs6988733 | 8 | 91535686 | T | C | CALB1---[]--TMEM64 | 0.0252 | 0.0063 |
| 182 | rs7006885 | 8 | 93283578 | A | G | RUNX1T1---[]---TRIQK | 0.0501 | 0.0066 |
| 183 | rs3100052 | 8 | 101967139 | A | G | YWHAZ-[]---ZNF706 | 0.0207 | 0.0061 |
| 184 | rs2737245 | 8 | 116658583 | T | G | [TRPS1] | 0.0347 | 0.0068 |
| 185 | rs1871729 | 8 | 136223702 | G | A | ZFAT---[]---KHDRBS3 | 0.0262 | 0.0064 |
| 186 | rs6477309 | 9 | 8450638 | T | C | [PTPRD] | 0.0348 | 0.0063 |
| 187 | rs2844016 | 9 | 24582747 | T | C | IZUMO3--[] | 0.0307 | 0.0066 |
| 188 | rs308521 | 9 | 37367094 | T | C | ZCCHC7-[]--GRHPR | 0.0307 | 0.0061 |
| 189 | rs4878734 | 9 | 38010085 | A | T | [SHB] | 0.0164 | 0.0060 |
| 190 | rs6560218 | 9 | 74245426 | C | T | TRPM3---[]--TMEM2 | 0.0209 | 0.0060 |
| 191 | rs62553781 | 9 | 76679777 | C | T | ANXA1---[]---RORB | 0.0597 | 0.0176 |
| 192 | rs12378543 | 9 | 83196097 | C | T | TLE4---[] | 0.0222 | 0.0062 |
| 193 | rs555784 | 9 | 85318704 | T | A | SPATA31D1---[]---RASEF | 0.0286 | 0.0062 |
| 194 | rs295268 | 9 | 86429305 | C | T | [GKAP1] | 0.0262 | 0.0068 |
| 195 | rs3138490 | 9 | 92219000 | A | T | SEMA4D---[]GADD45G | 0.0228 | 0.0060 |
| 196 | rs10759208 | 9 | 109806199 | C | T | ZNF462--[]---RAD23B | 0.0262 | 0.0062 |
| 197 | rs11788633 | 9 | 116767656 | C | G | [ZNF618] | 0.0292 | 0.0063 |
| 198 | rs10818834 | 9 | 126317324 | T | C | [DENND1A] | 0.0263 | 0.0067 |
| 199 | rs10988239 | 9 | 131943440 | C | T | IER5L-[]---C9orf106 | 0.0160 | 0.0062 |
| 200 | rs12380242 | 9 | 139310187 | C | T | [PMPCA] | 0.0161 | 0.0060 |
| 201 | rs28458909 | 9 | 140257189 | C | T | [EXD3] | 0.0719 | 0.0103 |
| 202 | rs497338 | 10 | 804315 | T | C | DIP2C--[]--LARP4B | 0.0292 | 0.0065 |
| 203 | rs66617308 | 10 | 56699338 | T | C | PCDH15---[]---MTRNR2L5 | 0.0169 | 0.0064 |
| 204 | rs9416744 | 10 | 60567937 | A | C | [BICC1] | 0.0443 | 0.0068 |
| 205 | rs11597421 | 10 | 61794146 | G | A | [ANK3] | 0.0262 | 0.0062 |
| 206 | rs12249410 | 10 | 64301941 | G | T | [ZNF365] | 0.0313 | 0.0098 |
| 207 | rs17712705 | 10 | 69623271 | G | A | DNAJC12--[]--SIRT1 | 0.0290 | 0.0064 |
| 208 | rs2298117 | 10 | 70346740 | C | T | [TET1] | 0.0180 | 0.0060 |
| 209 | rs10762434 | 10 | 73044413 | C | G | [UNC5B] | 0.0329 | 0.0071 |
| 210 | rs2648721 | 10 | 93026996 | G | T | [PCGF5] | 0.0211 | 0.0066 |
| 211 | rs61875203 | 10 | 93888810 | T | C | [CPEB3] | 0.0274 | 0.0067 |
| 212 | rs1163238 | 10 | 104943993 | G | A | [NT5C2] | 0.0184 | 0.0062 |
| 213 | rs7900191 | 10 | 119145774 | C | T | PDZD8--[]---EMX2 | 0.0183 | 0.0062 |
| 214 | rs11200159 | 10 | 123553392 | C | A | [ATE1] | 0.0226 | 0.0064 |
| 215 | rs3808964 | 10 | 125426627 | T | G | [GPR26] | 0.0154 | 0.0062 |
| 216 | rs9664044 | 10 | 126710791 | C | T | [CTBP2] | 0.0248 | 0.0071 |
| 217 | rs10830107 | 10 | 129304075 | A | G | DOCK1--[]--NPS | 0.0308 | 0.0072 |
| 218 | rs76518095 | 10 | 131149976 | T | C | []---MGMT | 0.0464 | 0.0117 |
| 219 | rs12771973 | 10 | 133749294 | G | A | [PPP2R2D] | 0.0277 | 0.0070 |
| 220 | rs60521023 | 11 | 13314102 | D | I | [ARNTL] | 0.0429 | 0.0066 |
| 221 | rs10832648 | 11 | 16618307 | C | A | SOX6---[]---C11orf58 | 0.0301 | 0.0074 |
| 222 | rs10742179 | 11 | 27650524 | A | G | LIN7C---[]--BDNF | 0.0385 | 0.0067 |
| 223 | rs4923541 | 11 | 28479535 | T | C | METTL15---[] | 0.0282 | 0.0060 |
| 224 | rs621421 | 11 | 30405914 | C | T | ARL14EP--[]--MPPED2 | 0.0289 | 0.0062 |
| 225 | rs11032362 | 11 | 33759092 | A | G | CD59--[]-FBXO3 | 0.0791 | 0.0101 |
| 226 | rs7111582 | 11 | 43893222 | G | A | HSD17B12--[]-ALKBH3 | 0.0478 | 0.0097 |
| 227 | rs10838687 | 11 | 47312892 | T | G | [MADD] | 0.0449 | 0.0074 |
| 228 | rs12808544 | 11 | 58373221 | C | A | [ZFP91] | 0.0342 | 0.0070 |
| 229 | rs662094 | 11 | 66342691 | A | G | CTSF-[]--CCDC87 | 0.0358 | 0.0060 |
| 230 | rs1278402 | 11 | 82972097 | A | G | ANKRD42--[]CCDC90B | 0.0380 | 0.0068 |
| 231 | rs1508608 | 11 | 92893825 | A | G | [SLC36A4] | 0.0402 | 0.0064 |
| 232 | rs4121878 | 11 | 95120372 | C | G | SESN3---[]---FAM76B | 0.0239 | 0.0060 |
| 233 | rs17577073 | 11 | 99152801 | A | C | [CNTN5] | 0.0280 | 0.0060 |
| 234 | rs2514214 | 11 | 113395329 | A | G | DRD2--[]---TMPRSS5 | 0.0257 | 0.0067 |
| 235 | rs4936290 | 11 | 114009255 | C | A | [ZBTB16] | 0.0189 | 0.0065 |
| 236 | rs3867239 | 11 | 122093090 | A | G | BLID---[]---UBASH3B | 0.0318 | 0.0061 |
| 237 | rs74357745 | 11 | 122811822 | A | G | [C11orf63] | 0.0213 | 0.0094 |
| 238 | rs7943634 | 11 | 126734319 | C | T | [KIRREL3] | 0.0204 | 0.0065 |
| 239 | rs3782860 | 12 | 361996 | T | C | [SLC6A13] | 0.0289 | 0.0060 |
| 240 | rs1799464 | 12 | 16286082 | G | A | DERA--[]--SLC15A5 | 0.0246 | 0.0067 |
| 241 | rs12298405 | 12 | 17015267 | C | T | LMO3---[] | 0.0236 | 0.0063 |
| 242 | rs2433634 | 12 | 23060363 | C | A | ETNK1---[]---SOX5 | 0.0279 | 0.0068 |
| 243 | rs11611435 | 12 | 24089322 | T | C | [SOX5] | 0.0225 | 0.0060 |
| 244 | rs13377754 | 12 | 34051765 | T | C | SYT10---[]---ALG10 | 0.0546 | 0.0061 |
| 245 | rs1843888 | 12 | 38737310 | A | G | ALG10B--[]---CPNE8 | 0.0601 | 0.0060 |
| 246 | rs247929 | 12 | 46294908 | C | G | [ARID2] | 0.0356 | 0.0060 |
| 247 | rs7975791 | 12 | 49413486 | T | C | [KMT2D] | 0.0536 | 0.0159 |
| 248 | rs4761989 | 12 | 52042142 | C | T | [SCN8A] | 0.0370 | 0.0087 |
| 249 | rs7299922 | 12 | 54702519 | A | G | NFE2-[]--COPZ1 | 0.0225 | 0.0064 |
| 250 | rs487722 | 12 | 57824165 | T | G | R3HDM2---[]-INHBC | 0.0256 | 0.0076 |
| 251 | rs10877962 | 12 | 63520912 | T | C | PPM1H---[]--AVPR1A | 0.0494 | 0.0061 |
| 252 | rs711098 | 12 | 77976559 | A | C | E2F7---[]---NAV3 | 0.0334 | 0.0061 |
| 253 | rs7959983 | 12 | 90452978 | C | T | ATP2B1---[]---CCER1 | 0.0283 | 0.0061 |
| 254 | rs7304278 | 12 | 106989915 | G | A | POLR3B--[]-RFX4 | 0.0296 | 0.0066 |
| 255 | rs7298532 | 12 | 112510404 | T | C | [NAA25] | 0.0252 | 0.0067 |
| 256 | rs3955311 | 12 | 114343818 | T | C | [RBM19] | 0.0314 | 0.0085 |
| 257 | rs80097534 | 12 | 121029604 | G | T | POP5--[]--CABP1 | 0.0239 | 0.0108 |
| 258 | rs9597241 | 13 | 56281271 | A | C | [] | 0.0365 | 0.0076 |
| 259 | rs12871550 | 13 | 59576365 | A | G | []---DIAPH3 | 0.0315 | 0.0064 |
| 260 | rs9571526 | 13 | 66590868 | G | T | []---PCDH9 | 0.0318 | 0.0071 |
| 261 | rs2593487 | 13 | 69903058 | G | A | []---KLHL1 | 0.0345 | 0.0064 |
| 262 | rs495593 | 13 | 72919800 | A | G | DACH1---[]---MZT1 | 0.0320 | 0.0067 |
| 263 | rs45597035 | 13 | 73649152 | G | A | [KLF5] | 0.0227 | 0.0063 |
| 264 | rs9573980 | 13 | 77590741 | A | G | [FBXL3] | 0.1498 | 0.0166 |
| 265 | rs1886205 | 13 | 94062095 | A | C | [GPC6] | 0.0337 | 0.0070 |
| 266 | rs9558942 | 13 | 107700218 | C | T | ARGLU1---[]---FAM155A | -0.0320 | 0.0063 |
| 267 | rs3815983 | 13 | 109779906 | C | T | [MYO16] | 0.0247 | 0.0063 |
| 268 | rs1163628 | 13 | 112226420 | C | A | TEX29---[]---SOX1 | 0.0331 | 0.0085 |
| 269 | rs61990287 | 14 | 42069889 | A | C | []-LRFN5 | 0.0268 | 0.0068 |
| 270 | rs2878172 | 14 | 55373670 | G | A | GCH1-[]--WDHD1 | 0.0296 | 0.0060 |
| 271 | rs962961 | 14 | 57281154 | C | T | OTX2-[]---EXOC5 | 0.0254 | 0.0063 |
| 272 | rs6573308 | 14 | 60806976 | T | C | PPM1A--[]--C14orf39 | 0.0263 | 0.0061 |
| 273 | rs7143933 | 14 | 62460219 | T | G | SNAPC1---[]-SYT16 | 0.0341 | 0.0068 |
| 274 | rs2978382 | 14 | 64769074 | T | C | ESR2-[]--MTHFD1 | 0.0211 | 0.0060 |
| 275 | rs4903203 | 14 | 74660508 | A | G | [LIN52] | 0.0267 | 0.0064 |
| 276 | rs12436039 | 14 | 79452019 | T | C | [NRXN3] | 0.0484 | 0.0092 |
| 277 | rs4550384 | 14 | 85350142 | T | G | []---FLRT2 | 0.0280 | 0.0071 |
| 278 | rs710284 | 14 | 98532540 | T | C | []---C14orf177 | 0.0247 | 0.0060 |
| 279 | rs11845599 | 14 | 101016824 | G | A | [BEGAIN] | 0.0343 | 0.0064 |
| 280 | rs59986227 | 15 | 48009263 | G | C | []-SEMA6D | 0.0293 | 0.0071 |
| 281 | rs12442008 | 15 | 53725112 | T | C | ONECUT1---[]--WDR72 | 0.0353 | 0.0067 |
| 282 | rs4775086 | 15 | 58969292 | G | A | [ADAM10] | 0.0299 | 0.0071 |
| 283 | rs12442674 | 15 | 96907819 | A | C | NR2F2--[]---SPATA8 | 0.0401 | 0.0080 |
| 284 | rs1873958 | 15 | 101147726 | A | G | [ASB7] | 0.0414 | 0.0061 |
| 285 | rs72773411 | 16 | 728514 | A | G | RHBDL1[]-STUB1 | 0.0353 | 0.0087 |
| 286 | rs12445235 | 16 | 8195278 | G | C | RBFOX1---[]---TMEM114 | 0.0239 | 0.0061 |
| 287 | rs2304467 | 16 | 8988777 | G | C | [USP7] | 0.0202 | 0.0063 |
| 288 | rs11641239 | 16 | 23124193 | T | C | [USP31] | 0.0174 | 0.0067 |
| 289 | rs7203707 | 16 | 24518569 | C | A | CACNG3---[]--RBBP6 | 0.0159 | 0.0060 |
| 290 | rs4785296 | 16 | 49467234 | C | G | C16orf78--[]--ZNF423 | 0.0292 | 0.0071 |
| 291 | rs3743794 | 16 | 52112312 | G | A | C16orf97-[]---TOX3 | 0.0205 | 0.0061 |
| 292 | rs12927162 | 16 | 52684916 | A | G | TOX3---[]---CHD9 | 0.0656 | 0.0068 |
| 293 | rs1421085 | 16 | 53800954 | C | T | [FTO] | 0.0439 | 0.0061 |
| 294 | rs2550298 | 16 | 56367969 | C | T | [GNAO1] | 0.0429 | 0.0063 |
| 295 | rs8044054 | 16 | 60628436 | T | C | [] | 0.0314 | 0.0061 |
| 296 | rs72790386 | 16 | 68136932 | T | G | [NFATC3] | 0.0709 | 0.0174 |
| 297 | rs17604349 | 16 | 72210865 | G | A | PMFBP1-[]---ZFHX3 | 0.0441 | 0.0075 |
| 298 | rs1061032 | 17 | 8064083 | T | G | [VAMP2] | 0.0647 | 0.0100 |
| 299 | rs11545787 | 17 | 17398278 | G | A | [RASD1] | 0.0705 | 0.0071 |
| 300 | rs12950382 | 17 | 30603994 | A | G | [RHBDL3] | 0.0270 | 0.0067 |
| 301 | rs4365329 | 17 | 31625887 | T | A | [ASIC2] | 0.0250 | 0.0060 |
| 302 | rs2011528 | 17 | 33980566 | C | T | [AP2B1] | 0.0294 | 0.0077 |
| 303 | rs3760381 | 17 | 43047083 | A | G | C1QL1-[]--DCAKD | 0.0301 | 0.0068 |
| 304 | rs7225002 | 17 | 44189067 | G | A | [KANSL1] | 0.0208 | 0.0061 |
| 305 | rs12600452 | 17 | 45054564 | A | G | GOSR2--[]RPRML | 0.0343 | 0.0075 |
| 306 | rs12051 | 17 | 46103760 | G | A | [COPZ2] | 0.0327 | 0.0061 |
| 307 | rs55846845 | 17 | 50092201 | G | A | [CA10] | 0.0210 | 0.0060 |
| 308 | rs72829706 | 17 | 54173733 | A | G | PCTP---[]--ANKFN1 | 0.0525 | 0.0152 |
| 309 | rs8072058 | 17 | 55734198 | T | A | [MSI2] | 0.0314 | 0.0072 |
| 310 | rs412000 | 17 | 56709058 | G | C | [TEX14] | 0.0250 | 0.0060 |
| 311 | rs58681483 | 17 | 57934654 | A | G | VMP1--[]-TUBD1 | 0.0551 | 0.0104 |
| 312 | rs72841368 | 17 | 61391114 | T | A | [TANC2] | 0.0303 | 0.0076 |
| 313 | rs2916148 | 17 | 65482109 | A | G | [PITPNC1] | 0.0332 | 0.0062 |
| 314 | rs2580160 | 18 | 1816036 | A | G | ADCYAP1---[]---METTL4 | 0.0242 | 0.0060 |
| 315 | rs62082402 | 18 | 5186566 | T | G | [C18orf42] | 0.0603 | 0.0111 |
| 316 | rs1788784 | 18 | 21159630 | G | A | [NPC1] | 0.0427 | 0.0063 |
| 317 | rs1013987 | 18 | 22630836 | C | T | HRH4---[]--ZNF521 | 0.0219 | 0.0061 |
| 318 | rs4419127 | 18 | 31663654 | A | G | [NOL4] | 0.0600 | 0.0063 |
| 319 | rs9950528 | 18 | 35762461 | G | A | CELF4---[] | 0.0251 | 0.0062 |
| 320 | rs12969848 | 18 | 38152835 | T | C | [] | 0.0363 | 0.0060 |
| 321 | rs9956387 | 18 | 44773382 | T | A | [SKOR2] | 0.0181 | 0.0060 |
| 322 | rs4800998 | 18 | 53429655 | A | T | TCF4---[]---TXNL1 | 0.0567 | 0.0078 |
| 323 | rs9964420 | 18 | 56824041 | C | A | [SEC11C] | 0.0640 | 0.0067 |
| 324 | rs11152350 | 18 | 60240352 | C | A | [ZCCHC2] | 0.0290 | 0.0060 |
| 325 | rs34329963 | 18 | 64526233 | C | T | CDH19---[]---DSEL | 0.0389 | 0.0095 |
| 326 | rs1025601 | 18 | 73056278 | C | T | TSHZ1--[]--SMIM21 | 0.0163 | 0.0061 |
| 327 | rs10402849 | 19 | 2695661 | T | C | [GNG7] | 0.0203 | 0.0075 |
| 328 | rs36055559 | 19 | 5799433 | G | A | DUS3L-[]--NRTN | 0.0455 | 0.0103 |
| 329 | rs7248205 | 19 | 10770305 | T | C | [ILF3] | 0.0318 | 0.0062 |
| 330 | rs9636202 | 19 | 18449238 | G | A | LSM4--[]-PGPEP1 | 0.0227 | 0.0068 |
| 331 | rs73026775 | 19 | 31052954 | G | A | ZNF536-[]---TSHZ3 | 0.0410 | 0.0104 |
| 332 | rs4804951 | 19 | 31673388 | A | G | ZNF536---[]--TSHZ3 | 0.0210 | 0.0064 |
| 333 | rs56113850 | 19 | 41353107 | C | T | [CYP2A6] | 0.0308 | 0.0061 |
| 334 | rs58876439 | 19 | 42600984 | A | G | [POU2F2] | 0.0531 | 0.0120 |
| 335 | rs11670534 | 19 | 47003906 | C | T | [PPP5D1] | 0.0261 | 0.0083 |
| 336 | rs6131805 | 20 | 16222093 | T | G | MACROD2---[]---KIF16B | 0.0259 | 0.0062 |
| 337 | rs6131942 | 20 | 17348608 | G | A | [PCSK2] | 0.0330 | 0.0060 |
| 338 | rs1474754 | 20 | 20077178 | G | A | [C20orf26] | 0.0289 | 0.0068 |
| 339 | rs6047481 | 20 | 21539564 | A | T | NKX2-2--[]---PAX1 | 0.0248 | 0.0064 |
| 340 | rs1737893 | 20 | 31051699 | C | T | [C20orf112] | 0.0279 | 0.0062 |
| 341 | rs2072727 | 20 | 43538733 | T | C | [PABPC1L] | 0.0334 | 0.0060 |
| 342 | rs57236847 | 20 | 44668401 | C | G | [SLC12A5] | 0.0297 | 0.0061 |
| 343 | rs695459 | 22 | 28848278 | C | T | [TTC28] | 0.0192 | 0.0061 |
| 344 | rs28459838 | 22 | 35846168 | T | C | MCM5--[]--RASD2 | 0.0321 | 0.0076 |
| 345 | rs118047999 | 22 | 38851675 | C | G | KCNJ4--[]--KDELR3 | 0.0363 | 0.0071 |
| 346 | rs139911 | 22 | 40704052 | C | T | [TNRC6B] | 0.0244 | 0.0060 |
| 347 | rs9611597 | 22 | 41864190 | A | T | [PHF5A] | 0.0400 | 0.0085 |
| 348 | rs6007594 | 22 | 45728370 | G | A | [FAM118A] | 0.0255 | 0.0067 |
| 349 | rs3747463 | X | 68751721 | C | T | [FAM155B] | 0.0316 | 0.0051 |
| 350 | rs213462 | X | 82704455 | C | A | []--POU3F4 | 0.0363 | 0.0050 |
| 351 | rs7060620 | X | 101012848 | T | A | ARMCX2--[]--NXF5 | 0.0449 | 0.0063 |

**Supplementary table 2: List of lead variants at P<5E-8 identified in the 23andMe morning person GWAS alone, adapted from Jones et al (2019).**

| **23 and Me Locus No** | **Lead Variant** | **Chr** | **Chr Position** | **Morning person allele** | **Non-morning person allele** | **Beta** | **SE** |
| --- | --- | --- | --- | --- | --- | --- | --- |
| 1 | rs1989147 | 1 | 7909373 | T | C | 0.0812 | 0.0079 |
| 2 | rs10927823 | 1 | 15993603 | G | A | 0.0374 | 0.0067 |
| 3 | rs10917509 | 1 | 19992066 | T | C | 0.0358 | 0.0063 |
| 4 | rs12752290 | 1 | 21535330 | C | T | 0.0383 | 0.0062 |
| 5 | rs12140153 | 1 | 62579891 | G | T | 0.0675 | 0.0121 |
| 6 | rs12040629 | 1 | 77705365 | A | G | 0.0835 | 0.0083 |
| 7 | rs1335720 | 1 | 91173988 | A | G | 0.04 | 0.0071 |
| 8 | rs12032173 | 1 | 96895804 | G | T | 0.0431 | 0.0078 |
| 9 | rs9436119 | 1 | 150467753 | A | G | 0.0525 | 0.0064 |
| 10 | rs1144566 | 1 | 182569626 | T | C | 0.3121 | 0.0187 |
| 11 | rs7522067 | 1 | 190071452 | G | A | 0.0428 | 0.0063 |
| 12 | rs7581403 | 2 | 2314997 | C | T | 0.0364 | 0.0066 |
| 13 | rs62124718 | 2 | 12822995 | G | A | 0.0569 | 0.0101 |
| 14 | rs1504155 | 2 | 49402250 | C | A | 0.0359 | 0.0061 |
| 15 | rs78986478 | 2 | 50515881 | G | C | 0.0692 | 0.0103 |
| 16 | rs1160648 | 2 | 56453588 | C | T | 0.0338 | 0.0062 |
| 17 | rs359246 | 2 | 60476639 | G | A | 0.0376 | 0.0061 |
| 18 | rs10201530 | 2 | 77256291 | T | C | 0.0401 | 0.0061 |
| 19 | rs34509802 | 2 | 101591710 | A | G | 0.0506 | 0.0081 |
| 20 | rs35125029 | 2 | 144261657 | G | A | 0.0537 | 0.0063 |
| 21 | rs6433478 | 2 | 175241482 | C | T | 0.0353 | 0.0062 |
| 22 | rs13427590 | 2 | 198516821 | T | C | 0.0676 | 0.0061 |
| 23 | rs4416211 | 2 | 215377588 | T | A | 0.0346 | 0.0061 |
| 24 | rs55886641 | 2 | 239319185 | T | C | 0.0923 | 0.0098 |
| 25 | rs62260792 | 3 | 50248427 | A | G | 0.0725 | 0.0114 |
| 26 | rs82825 | 3 | 52471942 | A | G | 0.0475 | 0.008 |
| 27 | rs7626335 | 3 | 71575177 | C | A | 0.0411 | 0.0065 |
| 28 | rs4077419 | 3 | 77188041 | C | A | 0.0469 | 0.0062 |
| 29 | rs56242353 | 3 | 82581831 | T | G | 0.0655 | 0.0095 |
| 30 | rs1449403 | 3 | 85591467 | A | G | 0.0545 | 0.0094 |
| 31 | rs9290069 | 3 | 160890946 | G | A | 0.0381 | 0.0061 |
| 32 | rs3850174 | 3 | 172364093 | T | A | 0.0386 | 0.0071 |
| 33 | rs9863471 | 3 | 174761963 | G | C | 0.04 | 0.0071 |
| 34 | rs1488137 | 3 | 182104957 | G | T | 0.0407 | 0.0061 |
| 35 | rs4698679 | 4 | 18262425 | C | T | 0.0379 | 0.0069 |
| 36 | rs4579124 | 4 | 105319081 | A | G | 0.0403 | 0.0068 |
| 37 | rs876684 | 4 | 130915946 | C | A | 0.0386 | 0.0063 |
| 38 | rs11731618 | 4 | 132506771 | G | T | 0.0456 | 0.0077 |
| 39 | rs35166798 | 4 | 156116677 | A | G | 0.0527 | 0.0095 |
| 40 | rs893191 | 5 | 59023325 | T | A | 0.0354 | 0.0062 |
| 41 | rs780405 | 5 | 87556339 | G | A | 0.0473 | 0.007 |
| 42 | rs12514961 | 5 | 106657285 | C | T | 0.036 | 0.0064 |
| 43 | rs1998528 | 6 | 12151610 | A | G | 0.037 | 0.0063 |
| 44 | rs62385532 | 6 | 13161394 | A | G | 0.0709 | 0.0067 |
| 45 | rs766406 | 6 | 26319588 | G | T | 0.0343 | 0.0064 |
| 46 | rs2653349 | 6 | 55142337 | A | G | 0.0738 | 0.0077 |
| 47 | rs1931805 | 6 | 62630863 | T | C | 0.0321 | 0.0061 |
| 48 | rs12195792 | 6 | 98705295 | A | T | 0.0471 | 0.0068 |
| 49 | rs76223855 | 6 | 153136141 | C | T | 0.2708 | 0.0274 |
| 50 | rs10237162 | 7 | 24085405 | T | C | 0.0495 | 0.0068 |
| 51 | rs1450870 | 7 | 32311726 | C | T | 0.0375 | 0.0062 |
| 52 | rs6969081 | 7 | 50624505 | A | T | 0.0462 | 0.0063 |
| 53 | rs10258689 | 7 | 96466047 | G | A | 0.0764 | 0.0076 |
| 54 | rs56269620 | 7 | 102489456 | G | A | 0.0512 | 0.0063 |
| 55 | rs73208648 | 7 | 114036921 | T | G | 0.0414 | 0.0076 |
| 56 | rs6968240 | 7 | 121942674 | A | C | 0.0331 | 0.0063 |
| 57 | rs4129572 | 7 | 133636888 | C | T | 0.0354 | 0.0062 |
| 58 | rs7463166 | 8 | 4833790 | G | A | 0.0471 | 0.0063 |
| 59 | rs35231275 | 8 | 9619909 | T | A | 0.0375 | 0.0068 |
| 60 | rs2736304 | 8 | 11236964 | C | T | 0.0354 | 0.0061 |
| 61 | rs16878810 | 8 | 31959274 | C | G | 0.0383 | 0.0067 |
| 62 | rs7842982 | 8 | 33731186 | G | T | 0.0382 | 0.0063 |
| 63 | rs62501026 | 8 | 53126512 | C | T | 0.0609 | 0.0084 |
| 64 | rs13251387 | 8 | 65014043 | G | A | 0.0345 | 0.0062 |
| 65 | rs72673538 | 8 | 93273270 | C | G | 0.0501 | 0.0068 |
| 66 | rs4279559 | 8 | 106220667 | G | A | 0.0442 | 0.008 |
| 67 | rs11787046 | 8 | 116682527 | T | A | 0.0389 | 0.007 |
| 68 | rs4948548 | 10 | 60570927 | G | A | 0.044 | 0.0068 |
| 69 | rs1351525 | 11 | 13301548 | A | T | 0.0435 | 0.0067 |
| 70 | rs10742179 | 11 | 27650524 | A | G | 0.0385 | 0.0069 |
| 71 | rs1222208 | 11 | 30361377 | A | C | 0.0403 | 0.0074 |
| 72 | rs1402954 | 11 | 33777334 | T | C | 0.0791 | 0.0102 |
| 73 | rs10838193 | 11 | 43898020 | G | A | 0.0352 | 0.0065 |
| 74 | rs11039155 | 11 | 47280762 | G | A | 0.0564 | 0.0088 |
| 75 | rs3107024 | 11 | 66202215 | C | T | 0.0375 | 0.0062 |
| 76 | rs1278402 | 11 | 82972097 | A | G | 0.038 | 0.0069 |
| 77 | rs12786007 | 11 | 92890392 | G | A | 0.0419 | 0.0066 |
| 78 | rs627858 | 11 | 114074031 | G | A | 0.0369 | 0.0065 |
| 79 | rs12310956 | 12 | 33970682 | A | G | 0.0571 | 0.0063 |
| 80 | rs6582579 | 12 | 38704819 | T | A | 0.0604 | 0.0061 |
| 81 | rs1650008 | 12 | 46298437 | G | A | 0.0376 | 0.0062 |
| 82 | rs10877962 | 12 | 63520912 | T | C | 0.0494 | 0.0063 |
| 83 | rs10778534 | 12 | 107500506 | C | T | 0.0381 | 0.0063 |
| 84 | rs1697497 | 13 | 55922259 | G | T | 0.041 | 0.0075 |
| 85 | rs4445791 | 13 | 59197211 | A | G | 0.0374 | 0.0061 |
| 86 | rs1407571 | 13 | 69871861 | A | T | 0.0362 | 0.0065 |
| 87 | rs9573980 | 13 | 77590741 | A | G | 0.1498 | 0.0169 |
| 88 | rs1890256 | 14 | 55881276 | T | C | 0.0407 | 0.007 |
| 89 | rs11158627 | 14 | 66889669 | T | C | 0.0371 | 0.0063 |
| 90 | rs10851460 | 15 | 48092994 | A | G | 0.0405 | 0.0071 |
| 91 | rs7174381 | 15 | 75613289 | C | A | 0.0375 | 0.0069 |
| 92 | rs1873958 | 15 | 101147726 | A | G | 0.0414 | 0.0062 |
| 93 | rs12927162 | 16 | 52684916 | A | G | 0.0656 | 0.0069 |
| 94 | rs55872725 | 16 | 53809123 | T | C | 0.044 | 0.0062 |
| 95 | rs2398144 | 16 | 56352854 | C | A | 0.0414 | 0.0062 |
| 96 | rs1013982 | 16 | 72450482 | A | G | 0.0375 | 0.0065 |
| 97 | rs35712131 | 16 | 76474219 | C | T | 0.034 | 0.0062 |
| 98 | rs1061032 | 17 | 8064083 | T | G | 0.0647 | 0.0102 |
| 99 | rs11545787 | 17 | 17398278 | G | A | 0.0705 | 0.0073 |
| 100 | rs11650615 | 17 | 46123698 | G | C | 0.0395 | 0.0067 |
| 101 | rs10853329 | 18 | 5194172 | T | C | 0.045 | 0.007 |
| 102 | rs1788784 | 18 | 21159630 | G | A | 0.0427 | 0.0064 |
| 103 | rs4419127 | 18 | 31663654 | A | G | 0.06 | 0.0064 |
| 104 | rs203179 | 18 | 38073467 | C | T | 0.0366 | 0.0061 |
| 105 | rs4800994 | 18 | 53403850 | T | C | 0.0582 | 0.008 |
| 106 | rs9964420 | 18 | 56824041 | C | A | 0.064 | 0.0068 |
| 107 | rs2425674 | 20 | 43529461 | C | G | 0.0335 | 0.0061 |
| 108 | rs133075 | 22 | 41081164 | T | G | 0.0364 | 0.0061 |

**Supplementary table 3: Demographics and lifestyle characteristics of a subset of morning and evening people who answered the mental health questions in all individuals and men only and women only.**

|  | **All individuals** | | | **Men Only** | | | **Women Only** | | |
| --- | --- | --- | --- | --- | --- | --- | --- | --- | --- |
|  | **Morning person (cases)** | **Evening person (controls)** | ***P^I^*** | **Morning person (cases)** | **Evening person (controls)** | ***P^I^*** | **Morning person (cases)** | **Evening person (controls)** | ***P^I^*** |
| *N* | 81,955 | 48,782 |  | 34,179 | 21,046 |  | 47,776 | 27,736 |  |
| *N* Male (%) | 34,179 (41.7) | 21, 046 (43.1) | 7.60E-04 |  |  |  |  |  |  |
| Mean age (SD) | 57.0 (7.5) | 55.7 (7.9) | <1.00E-15 | 57.7 (7.5) | 56.2 (8.0) | <1.00E-15 | 56.5 (7.5) | 55.4 (7.8) | <1.00E-15 |
| Mean BMI (SD) | 26.7 (4.5) | 27.0 (4.7) | <1.00E-15 | 27.3 (4.0) | 27.4 (4.1) | 3.30E-03 | 26.2 (4.8) | 26.7 (5.1) | <1.00E-15 |
| Mean TDI (SD) | -1.89 (2.7) | -1.61 (2.9) | 1.30E-10 | -1.97 (2.7) | -1.63 (2.9) | 5.60E-11 | -1.84 (2.7) | -1.61 (2.9) | 0.012 |
| Smoking status |  |  | <1.00E-15 |  |  | <1.00E-15 |  |  | <1.00E-15 |
| Never Smoker (%) | 48,441 (59.1) | 25,934 (53.2) |  | 18,424 (53.9) | 10,252 (48.7) |  | 30,017 (62.8) | 15,682 (56.5) |  |
| Former Smoker (%) | 28,863 (35.2) | 17,831 (36.6) |  | 13,428 (39.3) | 8,290 (39.4) |  | 15,435 (32.3) | 9,541 (34.4) |  |
| Current Smoker (%) | 3,938 (4.8) | 4,331 (8.9) |  | 1,973 (5.8) | 2,123 (10.1) |  | 1,965 (4.1) | 2,208 (8.0) |  |
| Missing (%) | 713 (0.9) | 686 (1.4) |  | 354 (1.0) | 381 (1.8) |  | 359 (0.8) | 305 (1.1) |  |
| Depressive symptoms (%) | 9,473 (11.6) | 6,643 (13.6) | <1.00E-15 | 2,963 (8.7) | 2,297 (10.9) | 8.10E-14 | 6,510 (13.6) | 4,346 (15.7) | 8.00E-11 |
| Major depression (%) | 18,232 (22.2) | 12,876 (26.4) | <1.00E-15 | 5,282 (15.5) | 4,084 (19.4) | <1.00E-15 | 12,950 (27.1) | 8,792 (31.7) | <1.00E-15 |
| Mean CIDI severity (SD) | 2.88 (2.9) | 3.25 (3.0) | <1.00E-15 | 2.15 (2.7) | 2.56 (2.8) | <1.00E-15 | 3.40 (3.0) | 3.77 (3.0) | <1.00E-15 |
| Mean PHQ9 severity (SD) | 2.63 (3.5) | 3.13 (4.0) | <1.00E-15 | 2.30 (3.4) | 2.80 (3.9) | <1.00E-15 | 2.86 (3.6) | 3.39 (4.1) | <1.00E-15 |
| Mean wellbeing score (SD) | 12.8 (1.9) | 12.4 (2.1) | <1.00E-15 | 12.8 (2.0) | 12.4 (2.0) | <1.00E-15 | 12.8 (1.9) | 12.4 (2.1) | <1.00E-15 |
| GAD (%) | 3,813 (4.7) | 2,824 (5.8) | 3.10E-12 | 1,260 (3.7) | 1,009 (4.8) | 5.50E-06 | 2,553 (5.3) | 1,815 (6.5) | 4.90E-08 |
| Mean GAD severity (SD) | 2.09 (3.3) | 2.32 (3.5) | 4.80E-10 | 1.68 (3.0) | 1.95 (3.3) | 5.50E-06 | 2.38 (3.5) | 2.61 (3.7) | 1.40E-05 |
| Composite Phase Deviation; CPD^II^ (SD) | 1.06 (0.7) | 1.13 (0.8) | <1.00E-15 | 1.07 (0.7) | 1.16 (0.8) | 1.90E-11 | 1.06 (0.7) | 1.12 (0.7) | 2.60E-08 |

^I^comparison of morning and evening people using logistic regression adjusted for age, sex (for all individuals), assessment centre, Townsend Deprivation Index (TDI), Body Mass Index (BMI), and smoking status.

^II^The actigraphy measure, CPD, was adjusted for age at actigraphy, season of actigraphy, sex (for all individuals), TDI, BMI and smoking status.

**Supplementary table 4: Demographics and lifestyle characteristics of morning and evening people in all unrelated individuals and in unrelated men only and women only.**

|  | **All individuals** | | | **Men only** | | | **Women only** | | |
| --- | --- | --- | --- | --- | --- | --- | --- | --- | --- |
|  | **Morning person (cases)** | **Evening person (controls)** | ***P^I^*** | **Morning person (cases)** | **Evening person (controls)** | ***P^I^*** | **Morning person (cases)** | **Evening person (controls)** | ***P^I^*** |
| *N* | 212,089 | 127,393 |  | 93,659 | 58,251 |  | 118,430 | 69,142 |  |
| *N* Male (%) | 44.2 | 45.7 | 7.50E-05 |  |  |  |  |  |  |
| Mean age (SD) | 57.8 (7.8) | 56.3 (8.2) | <1.00E-15 | 58.1 (7.9) | 56.4 (8.4) | <1.00E-15 | 57.5 (7.8) | 56.3 (8.1) | <1.00E-15 |
| Mean BMI (SD) | 27.3 (4.7) | 27.5 (4.9) | <1.00E-15 | 27.9 (4.2) | 27.8 (4.3) | 0.49 | 26.8 (5.0) | 27.3 (5.3) | <1.00E-15 |
| Mean TDI (SD) | -1.58 (2.9) | -1.32 (3.1) | 1.30E-09 | -1.57 (3.0) | -1.25 (3.1) | 3.90E-14 | -1.59 (2.9) | -1.38 (3.0) | 0.42 |
| Smoking status |  |  | <1.00E-15 |  |  | <1.00E-15 |  |  | <1.00E-15 |
| Never Smoker (%) | 119,047 (56.1) | 62,667 (49.2) |  | 47,014 (50.2) | 25,858 (44.4) |  | 72,033 (60.8) | 36,809 (53.2) |  |
| Former Smoker (%) | 74,559 (35.2) | 46,142 (36.2) |  | 36,921 (39.4) | 22,765 (39.1) |  | 37,638 (31.8) | 23,378 (33.8) |  |
| Current Smoker (%) | 16,038 (7.6) | 16,563 (13.0) |  | 8,437 (9.0) | 8,514 (14.6) |  | 7,601 (6.4) | 8,049 (11.6) |  |
| Missing (%) | 2,445 (1.2) | 2,021 (1.6) |  | 1,287 (1.4) | 1,114 (1.9) |  | 1,158 (0.98) | 906 (1.3) |  |
| Depressive symptoms (%) | 21,897 (10.3) | 16,005 (12.6) | <1.00E-15 | 7,554 (8.1) | 5,939 (10.2) | <1.00E-15 | 14,343 (12.1) | 10,066 (14.6) | <1.00E-15 |
| Major depression^II^ (%) | 15,487 (7.3) | 10,932 (8.6) | <1.00E-15 | 4,509 (4.8) | 3,482 (6.0) | 1.20E-15 | 10,978 (9.3) | 7,450 (10.8) | <1.00E-15 |
| Mean CIDI severity^II^ (SD) | 2.88 (2.9) | 3.25 (3.0) | <1.00E-15 | 2.15 (2.7) | 2.56 (2.8) | <1.00E-15 | 3.40 (3.0) | 3.77 (3.0) | <1.00E-15 |
| Mean PHQ9 severity^II^ (SD) | 2.63 (3.5) | 3.12 (4.0) | <1.00E-15 | 2.30 (3.4) | 2.79 (3.9) | <1.00E-15 | 2.87 (3.6) | 3.37 (4.1) | <1.00E-15 |
| Mean wellbeing score^II^ (SD) | 12.8 (1.9) | 12.4 (2.1) | <1.00E-15 | 12.8 (1.95) | 12.4 (2.0) | <1.00E-15 | 12.8 (1.9) | 12.4 (2.1) | <1.00E-15 |
| GAD^II^ (%) | 3,233 (1.5) | 2,423 (1.9) | 4.00E-11 | 1,069 (1.1) | 873 (1.5) | 6.60E-06 | 2,164 (1.8) | 1,550 (2.2) | 6.00E-07 |
| Mean GAD severity^II^ (SD) | 2.09 (3.3) | 2.31 (3.5) | 2.30E-07 | 1.68 (3.0) | 1.94 (3.3) | 1.50E-05 | 2.38 (3.5) | 2.60 (3.7) | 1.20E-03 |

^I^comparison of morning and evening people using logistic regression adjusted for age, sex (for all individuals), assessment centre, TDI, BMI and smoking status.

^II^available in up to 124,275 unrelated individuals that answered the UK Biobank follow-up mental health questionnaire.

|  |  |  | **Observational** | | **Genetic** | | **Genetic:IVW** | | **Genetic:Egger** | | | **Genetic:WM** | | **Genetic:PWM** | |
| --- | --- | --- | --- | --- | --- | --- | --- | --- | --- | --- | --- | --- | --- | --- | --- |
| **Outcome tested** | **Included individuals** | ***N^I^*** | **Odd ratio in morning people**  **(95% CI)** | ***P^II^*** | **OR^V^ 2-fold genetic increase in morningness (95% CI)** | ***P^III^*** | **OR^V^ 2-fold genetic increase in morningness (95% CI)** | ***P^IV^*** | **OR^V^ 2-fold genetic increase in morningness (95% CI)** | ***P^IV^*** | ***P_int*** | **OR^V^ 2-fold genetic increase in morningness (95% CI)** | ***P^IV^*** | **OR^V^ 2-fold genetic increase in morningness (95% CI)** | ***P^IV^*** |
| Depressive symptoms | All | 305,079 | 0.79 (0.77, 0.81) | <1.00E-15 | 0.92 (0.88, 0.97) | 1.00E-03 | 0.97 (0.95, 1.00) | 0.03 | 0.96 (0.91, 1.01) | 0.12 | 0.47 | 0.97 (0.95, 1,00) | 0.09 | 0.96 (0.93, 0.98) | 2.38E-03 |
|  | Men | 146,334 | 0.79 (0.76, 0.82) | <1.00E-15 | 0.93 (0.85, 1.00) | 0.06 | 0.99 (0.96, 1.03) | 0.71 | 0.97 (0.90, 1.05) | 0.46 | 0.52 | 0.97 (0.93, 1.02) | 0.22 | 0.99 (0.93, 1.03) | 0.49 |
|  | Women | 158,745 | 0.79 (0.77, 0.81) | <1.00E-15 | 0.91 (0.86, 0.97) | 3.00E-03 | 0.97 (0.94, 0.99) | 0.01 | 0.95 (0.89, 1.01) | 0.11 | 0.54 | 0.95 (0.91, 0.98) | 3.58E-03 | 0.94 (0.90, 0.97) | 6.22E-04 |
| Major depression | All | 121,384 | 0.84 (0.82, 0.86) | <1.00E-15 | 0.95 (0.90, 1.01) | 0.10 | 0.98 (0.95, 1.00) | 0.06 | 1.01 (0.95, 1.08) | 0.78 | 0.24 | 1.00 (0.96, 1.03) | 0.84 | 1.00 (0.95, 1.03) | 0.83 |
|  | Men | 51,858 | 0.82 (0.78, 0.86) | <1.00E-15 | 0.88 (0.79, 0.98) | 0.02 | 0.97 (0.90, 1.05) | 0.46 | 1.20 (0.95, 1.51) | 0.12 | 0.06 | 0.96 (0.86, 1.08) | 0.51 | 0.96 (0.82, 1.08) | 0.48 |
|  | Women | 69,526 | 0.85 (0.82, 0.88) | <1.00E-15 | 0.99 (0.93, 1.05) | 0.75 | 0.94 (0.88, 1.00) | 0.06 | 0.94 (0.78, 1.13) | 0.53 | 0.99 | 0.95 (0.87, 1.03) | 0.21 | 0.95 (0.85, 1.03) | 0.22 |
| CIDI severity | All | 130,737 | 0.85 (0.83, 0.86) | <1.00E-15 | 0.97 (0.93, 1.01) | 0.13 | 0.95 (0.92, 0.98) | 4.09E-03 | 1.04 (0.96, 1.14) | 0.33 | 0.02 | 0.96 (0.91, 1.00) | 0.05 | 0.97 (0.91, 1.02) | 0.21 |
|  | Men | 55,225 | 0.83 (0.80, 0.85) | <1.00E-15 | 0.94 (0.88, 1.01) | 0.11 | 0.94 (0.86, 1.02) | 0.15 | 1.25 (0.97, 1.61) | 0.08 | 0.02 | 0.96 (0.85, 1.08) | 0.50 | 0.96 (0.82, 1.08) | 0.48 |
|  | Women | 75,512 | 0.86 (0.84, 0.88) | <1.00E-15 | 0.98 (0.94, 1.03) | 0.52 | 0.89 (0.81, 0.97) | 0.01 | 1.21 (0.93, 1.57) | 0.15 | 0.01 | 0.96 (0.86, 1.08) | 0.52 | 0.98 (0.83, 1.10) | 0.71 |
| PHQ9 severity | All | 130,737 | 0.85 (0.84, 0.87) | <1.00E-15 | 0.97 (0.93, 1.01) | 0.10 | 0.95 (0.90, 0.99) | 0.02 | 1.01 (0.90, 1.13) | 0.92 | 0.25 | 0.96 (0.90, 1.02) | 0.16 | 0.96 (0.88, 1.02) | 0.18 |
|  | Men | 55,225 | 0.84 (0.82, 0.87) | <1.00E-15 | 0.92 (0.86, 0.99) | 0.02 | 0.89 (0.80, 1.00) | 0.06 | 1.03 (0.73, 1.44) | 0.87 | 0.39 | 0.88 (0.75, 1.02) | 0.10 | 0.88 (0.72, 1.03) | 0.12 |
|  | Women | 75,512 | 0.86 (0.84, 0.88) | <1.00E-15 | 0.99 (0.95, 1.04) | 0.83 | 0.92 (0.82, 1.03) | 0.13 | 1.12 (0.80, 1.57) | 0.51 | 0.22 | 0.93 (0.80, 1.08) | 0.32 | 0.92 (0.76, 1.07) | 0.30 |
|  |  |  |  |  |  |  |  |  |  |  |  |  |  |  |  |
| Wellbeing | All | 127,236 | 1.29 (1.27, 1.32) | <1.00E-15 | 1.05 (1.01, 1.10) | 0.01 | 1.06 (1.03, 1.08) | 1.89E-05 | 1.00 (0.94, 1.06) | 0.90 | 0.03 | 1.03 (1.00, 1.07) | 0.05 | 1.03 (0.98, 1.06) | 0.09 |
|  | Men | 53,617 | 1.34 (1.30, 1.38) | <1.00E-15 | 1.05 (0.98, 1.13) | 0.14 | 1.13 (1.06, 1.20) | 1.39E-04 | 1.01 (0.84, 1.21) | 0.96 | 0.19 | 1.12 (1.03, 1.23) | 0.01 | 1.11 (0.98, 1.21) | 0.03 |
|  | Women | 73,619 | 1.25 (1.22, 1.28) | <1.00E-15 | 1.06 (1.00, 1.11) | 0.03 | 1.11 (1.05, 1.18) | 6.92E-04 | 1.00 (0.84, 1.20) | 0.99 | 0.23 | 1.13 (1.05, 1.22) | 1.48E-03 | 1.11 (1.00, 1.20) | 0.01 |
|  |  |  |  |  |  |  |  |  |  |  |  |  |  |  |  |
| GAD | All | 86,464 | 0.83 (0.79, 0.87) | 2.70E-12 | 0.93 (0.84, 1.03) | 0.18 | 0.99 (0.94, 1.04) | 0.77 | 0.99 (0.87, 1.11) | 0.83 | 0.92 | 1.01 (0.94, 1.08) | 0.76 | 1.00 (0.91, 1.08) | 0.93 |
|  | Men | 40,157 | 0.82 (0.75, 0.89) | 5.50E-06 | 0.97 (0.80, 1.18) | 0.78 | 1.00 (1.00, 1.01) | 0.43 | 1.02 (1.00, 1.05) | 0.04 | 0.06 | 1.01 (1.00, 1.02) | 0.10 | 1.00 (0.98, 1.01) | 0.46 |
|  | Women | 46,307 | 0.84 (0.78, 0.89) | 8.20E-08 | 0.91 (0.81, 1.03) | 0.14 | 1.00 (0.89, 1.12) | 0.98 | 1.00 (0.71, 1.41) | 0.99 | 0.98 | 0.96 (0.82, 1.12) | 0.59 | 0.95 (0.76, 1.13) | 0.59 |
| GAD severity | All | 130,232 | 0.92 (0.90, 0.94) | <1.00E-15 | 0.98 (0.94, 1.03) | 0.45 | 1.01 (0.97, 1.05) | 0.78 | 1.01 (0.92, 1.12) | 0.79 | 0.87 | 0.98 (0.93, 1.04) | 0.53 | 0.97 (0.90, 1.02) | 0.26 |
|  | Men | 55,056 | 0.91 (0.88, 0.94) | 2.10E-08 | 0.96 (0.89, 1.03) | 0.26 | 1.03 (0.94, 1.14) | 0.51 | 1.07 (0.80, 1.42) | 0.67 | 0.82 | 1.08 (0.95, 1.23) | 0.26 | 1.08 (0.89, 1.24) | 0.29 |
|  | Women | 75,176 | 0.92 (0.90, 0.95) | 3.00E-09 | 1.00 (0.95, 1.05) | 0.99 | 1.01 (0.91, 1.11) | 0.88 | 1.16 (0.86, 1.56) | 0.32 | 0.32 | 0.98 (0.85, 1.12) | 0.74 | 0.96 (0.81, 1.10) | 0.59 |

**Supplementary table 5: Associations between diurnal preference and mental health and wellbeing using regression models and 1 sample and 2 sample mendelian randomization methods in up to 305,079 UKBiobank participants.**

^I^*N* represents the number of UKBiobank participants that have both information on diurnal preference and the mental health outcome available.

^II^observational analysis using logistic regression (binary) and ordered logistic regression (continuous), adjusted for age, sex, centre, BMI, TDI and smoking status.

^III^using one-sample mendelian randomization methods, adjusted for age, sex, centre, chip and principle components(1-5).

^IV^ Using two-sample Mendelian Randomization methods, adjusted for age, sex, centre and chip. The inverse-variance weighted approach is used as the main analysis which assumes no horizontal pleiotropy. MR-Egger, WM and PWM have been used as sensitivity analysis, and providing all methods yield similar findings we can be more confident when suggesting causal inference.

^V^OR represents a doubling in the genetic risk of morningness see methods for more detail.

|  |  |  | **Observational** | | **Genetic** | | **Genetic:IVW** | | **Genetic:Egger** | | | **Genetic:WM** | | **Genetic:PWM** | |
| --- | --- | --- | --- | --- | --- | --- | --- | --- | --- | --- | --- | --- | --- | --- | --- |
| **Outcome tested** | **Included individuals** | ***N*** | **𝛽 (95% CI)** | ***P^I^*** | **𝛽^IV^ per doubling in the genetic risk for morningness (95% CI)** | ***P^II^*** | **𝛽^IV^ per doubling in the genetic risk for morningness (95% CI)** | ***P^III^*** | **𝛽^IV^ per doubling in the genetic risk for morningness (95% CI)** | ***P^III^*** | ***P_int*** | **𝛽^IV^ per doubling in the genetic risk for morningness (95% CI)** | ***P^III^*** | **𝛽^IV^ per doubling in the genetic risk for morningness (95% CI)** | ***P^III^*** |
| CPD | All | 76,334 | -0.05 (-0.06, -0.04) | <1.00E-15 | -0.02 (-0.06, 0.02) | 0.39 | -0.04 (-0.07, -0.01) | 3.37E-03 | -0.02 (-0.10, 0.06) | 0.63 | 0.60 | -0.05 (-0.08, -0.02) | 2.96E-03 | -0.05 (-0.08, -0.01) | 5.78E-03 |

**Supplementary table 6: Associations between diurnal preference and circadian misalignment using linear regression and one and two sample mendelian randomization methods.**

^I^ observational analysis using linear regression adjusted for age at actigraphy, season of actigraphy and sex

^II^using one sample mendelian randomization adjusted for principle components (1-5), age at actigraphy, sex, chip and season of actigraphy.

^III^ using two sample mendelian randomization methods.

^IV^Here we present the 𝛽 coefficient which represents the average change in CPD per doubling in the genetic risk for morningness see methods for more detail.

**Supplementary table 7: Observational associations between behavioural circadian misalignment and mental health and wellbeing using logistic regression (binary) and ordered logistic regression (continuous) models.**

|  | **CPD** | |
| --- | --- | --- |
| **Outcome tested** | **OR (95% CI)** | ***P^I^*** |
| Depressive symptoms | 1.20 (1.17, 1.23) | <1.00E-15 |
| Major depression | 1.19 (1.16, 1.21) | <1.00E-15 |
| CIDI severity | 1.17 (1.15, 1.19) | <1.00E-15 |
| PHQ9 severity | 1.22 (1.20, 1.23) | <1.00E-15 |
|  |  |  |
| Wellbeing | 0.89 (0.88, 0.91) | <1.00E-15 |
|  |  |  |
| GAD | 1.30 (1.25, 1.35) | <1.001E-15 |
| GAD severity | 1.11 (1.09, 1.12) | <1.00E-15 |

^I^adjusted for age at actigraphy, season of actigraphy and sex

Supplementary figure 1: A DAG representing the presumed complex relationship between diurnal preference, CPD and mental health.

Here we represent the environmental component of the G*E (U2) independent of other confounders as there is no evidence for e.g. age/sex adjusted diurnal preference to be influenced by work schedule, nor do we see sleep timing on free days (chronotype measured by sleep timing) significant and big differences in sleep timing as a function of preceding days(8).

****Supplementary figure 2: The observational and genetic associations between increased morningness and a) depressive symptoms, major depression, depression severity and current depression severity b) wellbeing c) generalised anxiety and anxiety severity in all individuals and in men and women separately (with eveningness as referent).

Supplementary figure 3: Plot of the individual diurnal preference variant-chronotype associations against the diurnal preference variant-(a)depressive symtoms-(b)wellbeing-(c)GAD associations on natural log scale (LN(OR)).The beta regression coefficients for inverse variance weighted (IVW) instrumental analysis (blue), Egger-MR (red), median-IV (pink) and the penalized weight median IV (green) are plotted.

c.


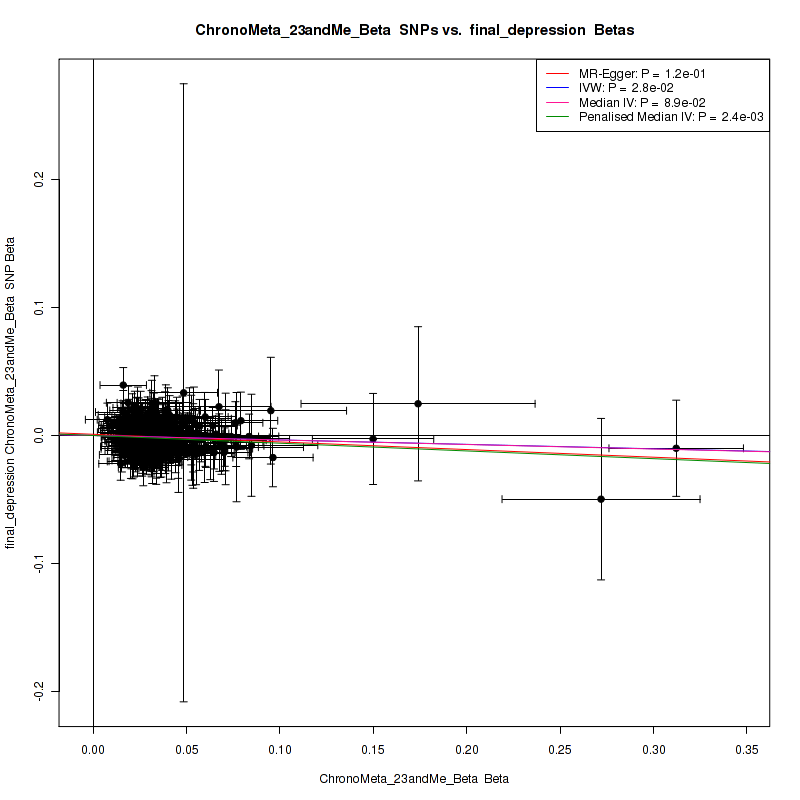

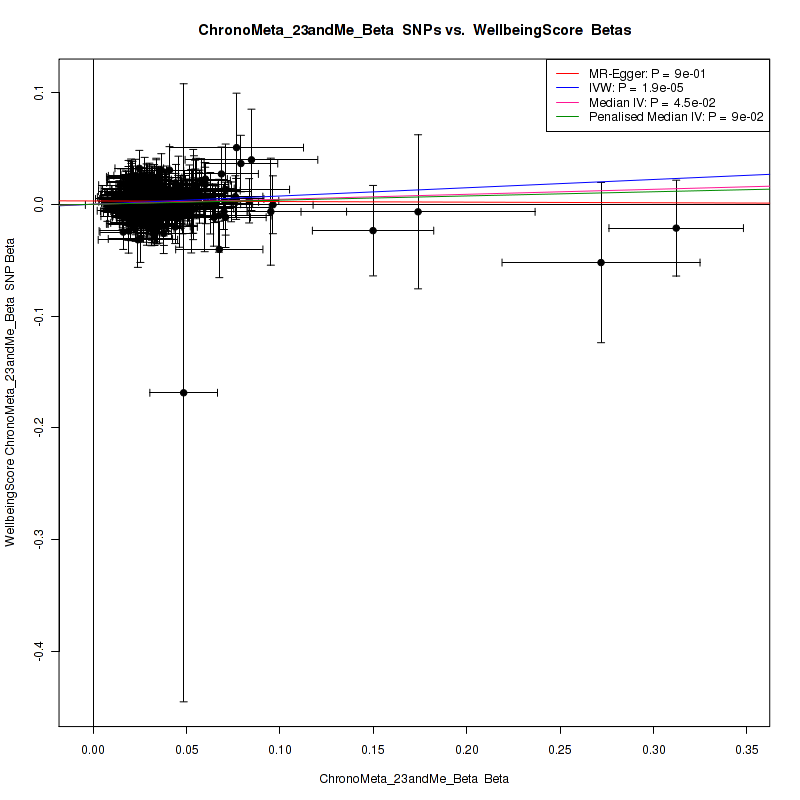

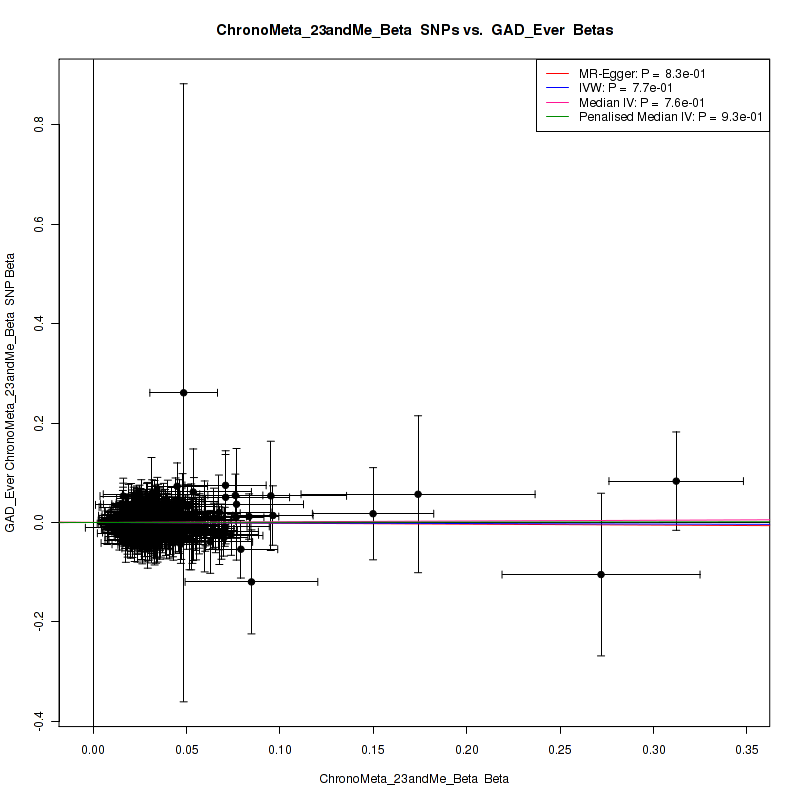


a.

b.

****Supplementary figure 4: The two-sample MR IVW ln(OR) in the full SNP list (*N=*351) and the 23andMe-only identified SNPs (*N=*108).

****Supplementary figure 5: The observational and genetic associations between increased morningness and (a) depression outcomes, (b) wellbeing, and (c) anxiety outcomes in all individuals and shift workers and non-shift workers individually (with eveningness as referent).

****Supplementary figure 6: The observational associations between morningness and lifetime depression severity, current depression severity, anxiety and anxiety severity with any individuals that reported changes in sleep patterns or trouble falling asleep excluded (with eveningness as referent).

Supplementary figure 7: The observational associations between behavioural circadian misalignment and mental health outcomes stratified by sex. Logistic regression models were used to produce odds ratios per standard deviation increase in misalignment adjusted for age at actigraphy and season at actigraphy.

Supplementary figure 8: The observational associations between behavioural circadian misalignment and mental health outcomes stratified by age above and below 65 years. We have included all individuals, individuals younger than 65 and individuals aged 65+ to investigate misalignment in those who are retired and those who are not. Retirement age has been presumed at 65 years.

Supplementary figure 9: The observational associations between behavioural circadian misalignment and mental health outcomes further adjusted for diurnal preference and the diurnal preference GRS, separately.

Supplementary figure 10: The observational associations between behavioural circadian misalignment and mental health outcomes in all individuals and shift workers and non-shift workers individually.

Supplementary figure 11: The observational associations between all day midsleep CPD, ‘free day’ midsleep CPD and the seven mental health and wellbeing outcomes.

Supplementary figure 12:The observational associations between behavioural circadian misalignment and mental health and wellbeing in all individuals and those taking relevant medications and not taking medication separately. Relevant medications included sleep medications, antidepressants, antipsychotics and anxiolytics. A full description can be found in the supplementary methods.

**References**

1. van Hees VT, Sabia S, Anderson KN, Denton SJ, Oliver J, Catt M, et al. A Novel, Open Access Method to Assess Sleep Duration Using a Wrist-Worn Accelerometer. PLOS ONE. 2015;10(11):e0142533.

2. Jairo HM, Alex VR, Florian H, Séverine S, Vincent TvH. GGIR: A Research Community–Driven Open Source R Package for Generating Physical Activity and Sleep Outcomes From Multi-Day Raw Accelerometer Data. Journal for the Measurement of Physical Behaviour. 2019;2(3):188-96.

3. van Hees VT, Sabia S, Jones SE, Wood AR, Anderson KN, Kivimäki M, et al. Estimating sleep parameters using an accelerometer without sleep diary. Scientific Reports. 2018;8(1):12975.

4. Fischer D, Vetter C, Roenneberg T. A novel method to visualise and quantify circadian misalignment. Sci Rep. 2016;6:38601-.

5. Fischer D, Vetter C, Roenneberg T. A novel method to visualise and quantify circadian misalignment. Scientific Reports. 2016;6(1):38601.

6. Adams MJ, Hill WD, Howard DM, Dashti HS, Davis KAS, Campbell A, et al. Factors associated with sharing e-mail information and mental health survey participation in large population cohorts. Int J Epidemiol. 2019.

7. Swinson RP. The GAD-7 scale was accurate for diagnosing generalised anxiety disorder. Evid Based Med. 2006;11(6):184.

8. Juda M, Vetter C, Roenneberg T. The Munich ChronoType Questionnaire for Shift-Workers (MCTQShift). J Biol Rhythms. 2013;28(2):130-40.
